# Identification and in vitro and in vivo validation of the key role of GSDME in pyroptosis-related genes signature in hepatocellular carcinoma

**DOI:** 10.1186/s12885-023-10850-1

**Published:** 2023-05-06

**Authors:** Xinyi Chen, Mu Yang, Lu Wang, Yuan Wang, Jingyao Tu, Xiao Zhou, Xianglin Yuan

**Affiliations:** Department of Oncology, Tongji Hospital, Tongji Medical College, Huazhong University of Science and Technology, Jie Fang Road 1095, Wuhan, Hubei China

**Keywords:** Pyroptosis, GSDME, Liver hepatocellular carcinoma (LIHC), Prognostic signature, Bioinformatics analysis

## Abstract

We used pyroptosis-related genes to establish a risk–score model for prognostic prediction of liver hepatocellular carcinoma (LIHC) patients. A total of 52 pyroptosis-associated genes were identified. Then, data for 374 LIHC patients and 50 normal individuals were acquired from the TCGA database. Through gene expression analyses, differentially expressed genes (DEGs) were determined. The 13 pyroptosis-related genes (PRGs) confirmed as potential prognostic factors through univariate Cox regression analysis were entered into Lasso and multivariate Cox regression to build a PRGs prognostic signature, containing four PRGs (BAK1, GSDME, NLRP6, and NOD2) determined as independent prognostic factors. mRNA levels were evaluated by qRT-PCR, while overall survival (OS) rates were assessed by the Kaplan–Meier method. Enrichment analyses were done to establish the mechanisms associated with differential survival status of LIHC patients from a tumor immunology perspective. Additionally, a risk score determined by the prognostic model could divide LIHC patients into low- and high-risk groups using median risk score as cut-off. A prognostic nomogram, derived from the prognostic model and integrating clinical characteristics of patients, was constructed. The prognostic function of the model was also validated using GEO, ICGC cohorts, and online databases Kaplan–Meier Plotter. Small interfering RNA-mediated knockdown of GSDME, as well as lentivirus-mediated GSDME knockdown, were performed to validate that knockdown of GSDME markedly suppressed growth of HCC cells both in vivo and in vitro. Collectively, our study demonstrated a PRGs prognostic signature that had great clinical value in prognosis assessment.

**Supplementary Information:**

The online version contains supplementary material available at 10.1186/s12885-023-10850-1.

## Introduction

Hepatocellular carcinoma (HCC) is known to represent 85–90% of primary liver cancer cases, which is the 6th prevalent tumor, and the 4th major contributor to tumor-associated death globally. Clinical incidences, as well as mortality rates of HCC patients, are increasing year by year [1, 2]. Thus, there is an urgent need for identifying effective markers to inform the individualized treatment of HCC patients.

Given rapid advances in bioinformatic analytics, prognostic signature based on various newly defined cell death mechanisms-associated genes is increasingly being used as novel indicators for development and progression of cancer, such as ferroptosis [3], pyroptosis [4] and cuproptosis [5]. Pyroptosis, triggered by pro-inflammatory signals and also denoted as cell inflammatory necrosis, is postulated as gasdermin-induced programmed necrosis [6]. Pyroptosis plays various roles in several processes in diseases such as cardiovascular diseases [7], Alzheimer’s disease [8], diabetes [9], Parkinson’s disease [10], autoimmune diseases [11], and so on. Most noteworthy, the significance of pyroptosis in the incidence and progression of cancer is being evaluated [12]. Several inflammatory mediators, produced as a result of the activation of signaling pathways, play a role in carcinogenesis during pyroptosis. For instance, PRGs, including NLRP3 [13], Gasdermin D (GSDMD) [14], Caspase 1 (CASP1) [15], and Gasdermin E (GSDME) [16], are highly associated with oncogenesis as well as tumor progression. Additionally, Zheng et al. confirmed that STAT3β dysregulated mitochondrial electron transport chain promoted chemosensitivity by initiating pyroptosis in esophageal squamous cell carcinoma [17]. NLRP1, a component of pyroptosis, facilitates caspase-1-dependent IL-1b as well as IL-18 secretion, thereby promoting skin cancer [18]. A large amount of data also suggests that pyroptosis has crosstalk with the tumor immune microenvironment [19]. In summary, a combined study of pyroptosis and LIHC is urgently needed due to the fact that pyroptosis performs such a crucial function in cancer and that there has been limited research addressing its involvement in the incidence and progression of LIHC so far.

It is worth mentioning that complex tumor microenvironment (TME), especially tumor immune microenvironment (TIME), has been suggested as potential prognostic factors affecting clinical outcome in patients with malignancies [20]. Additionally, the TIME composed of tumor‐infiltrating immune cells is found in close association with various approaches for tumor treatment, including chemoradiotherapy, molecular-targeted therapies, and immunotherapy [21]. It is becoming increasingly popular in bioinformatics to identify markers involved in regulating TME and TIME, with immune-related prognostic genes being of particular interest [22, 23]. In particular, prognostic signatures can also serve as efficacy indicators of anti-tumor drugs. Overall, identifying sensitive and specific biomarkers may facilitate LIHC treatment and diagnosis.

In this work, we obtained LIHC RNA sequencing data from TCGA, GEO as well as ICGC databases and then developed an effective prognostic model of pyroptosis-related biomarkers to explore potential mechanisms and clinical significance for LIHC. Above all, we discovered that high GSDME expression indicated a more advanced stage with poorer prognosis of LIHC patients.

## Materials and methods

### Data collection

RNA sequence data (Illumina HiSeq RNA-Seq platform) and the matching clinical data for LIHC patients were retrieved from The Cancer Genome Atlas (TCGA) database. There were 374 cancer samples and 50 normal tissue samples in the LIHC cohort. After retrieving the Gene Expression Omnibus (GEO) database (http://www.ncbi.nlm.nih.gov/geo/), three HCC datasets GSE62232 [24], GSE102079 [25] and GSE112790 [26] (containing 91, 166 and 198 samples, respectively) were selected for bioinformatics analysis (involving analyzing BAK1, GSDME and NLRP6 levels in cancer tissues, compared to non-cancer tissues). The Illumina HiSeq RNA Seq-based LIHC-related gene expression files (ICGC-LIRI-JP), involving 442 Japanese patients, were acquired from the international cancer genomics consortium (ICGC, https://icgc.org/). Next, 52 pyroptosis-associated genes (Supplementary Table S1) were obtained from previous research and literature [27–29], which has proteins and the equivalent coding genes of several species involved in cellular death.

### Patients

The sections from fourteen patients with HCC were obtained from the Department of Pathology in Tongji Hospital, China. Immunohistochemically GSDME antibody was applied to the tumor areas. All experiments were approved by the clinical ethics committee of Huazhong University of Science and Technology.

### Determination of differentially expressed PRGs

“Edger” in R was used to normalize read count values. DEGs between the normal and tumor groups were also subjected to an evaluation with the aid of the "limma" software package [30], with a false discovery rate (FDR) < 0.05 and |log2FC|≥ 1.

### Development of pyroptosis-related genes prognostic signature

The clinical-pathological data, such as survival time, Stage, Grade, survival status, age, gender, as well as TNM classification, were acquired from TCGA-LIHC. Univariate Cox regression analyses were first performed for the purpose of screening the PRGs that were prognosis-associated in LIHC (*p* < 0.05). Then, multivariable Cox regression analyses were conducted to develop a prognostic model by integrating all independent predictors identified through Lasso regression analyses. Each gene’s risk coefficient was also determined utilizing R package “glmnet” after narrowing down the genes with overfitting risk, according to the following formula:

$$riskscore=\sum_{i=1}^{n}coefficient\left(i\right)*expression\left(i\right).$$

Additionally, after setting the median risk score as a threshold criterion, LIHC patients were assigned a greater risk value (50%) or a lower risk value (50%). They were classified into low- and high-risk groups. The receiver operating characteristic (ROC) curve was generated utilizing the R package "survival ROC" with the aim of determining the prediction power of the constructed prognostic model. Prognostic analysis was conducted with the help of the R program "survival", and Kaplan–Meier plots with the log-rank test were plotted with the aid of the R package "survplot". According to the four PRGs, we performed principal component analysis (PCA) with the “prcomp” function in “stats” R package. In order to prove that the PRGs prognostic model remained independent of other clinical-pathological parameters, we conducted Lasso regression, multivariate Cox regression as well as univariate Cox regression analyses. Subsequently, we created the nomogram based on these models. Through the use of the calibration curve, we examined whether the anticipated probabilities from the nomogram were consistent with the real recorded outcome. The heatmap was created using the R package "pheatmap" in order to provide a more comprehensible and visual representation of the difference in the expression levels of PRG between the two risk groups, as well as the clinical and pathological indices.

### Molecular mechanism and immune infiltrate analysis

ClusterProfiler was utilized for PRGs functional annotation analysis to comprehensively investigate the functional correlations between these prognostic genes [31]. We analyzed the functions of differentially expressed PRGs by GO and KEGG [32] with “clusterProfiler” package (FDR < 0.05). In the meantime, the TIMER [33], CIBERSORT [34], CIBERSORT − ABS [35], QuanTIseq [36], MCPcounter [37], xCell [38], and Epic [39] algorithms were conducted to estimate cellular constituents or immunological responsiveness between low- and high-risk groups according to the PRGs prognostic signature. Through the use of a heatmap, we successfully visualized the differences in immunological responses through various algorithms. Additionally, the single-sample gene set enrichment analysis (ssGSEA) was performed for the purpose of quantifying the tumor-infiltrating immune cell subgroups between the 2 groups and determining their immunological activity. Previous research was also used to identify possible immune checkpoints.

### Consensus clustering analysis of PRGs

Patients were divided into several subgroups utilizing the "ConsensusClusterPlus" tool as a resampling-based technique with repeated computation for a total of 1000 times in order to analyze the biological characteristics of PRGs in patients with LIHC [40]. Variations in gene expression levels in various LIHC subtypes were determined by PCA.

### The Human Protein Atlas (HPA)

HPA, an online database, contains cell-, tissue-, and organ-derived human protein data [41]. Immunohistochemistry data of the PRGs in prognostic signature between HCC and normal liver tissues were retrieved from this database.

### Cell culture and treatment

Human hepatoma cell lines (SK-Hep1, HepG2, and HuH-7), as well as the human hepatocyte LO2 cell line, were acquired from the America Type Culture Collection (ATCC, Manassas, VA, United States). Cell incubation was performed in a humid 5% CO2 environment at 37 °C. LO2 cells were preserved in RPMI 1640 medium that contained fetal bovine serum (10%, FBS). HepG2, HuH-7, and SK-Hep1 cells were cultured in 10% FBS-supplemented DMEM (GIBCO-BRL, Thermo Fisher Scientific, Waltham, MA, United States).

### Western blotting analysis

Total cellular proteins were extracted using RIPA buffer that contained 1% PMSF. The total protein was evaluated by BCA protein assay kits (BOSTER, Wuhan, China) following the manufacturer's protocol. Afterward, the proteins were separated using electrophoresis. Following electrophoresis, isolated proteins were subjected to transferring to polyvinylidene fluoride (PVDF) membrane and blocking by incubation in the presence of 5% non-fat skim milk in TBS-T buffer, followed by overnight incubation with antibody anti-GSDME (1:1000; catalog number A7432; ABclonal, Wuhan, China) and anti-GAPDH (1:5000, AC002, Wuhan, China) at 4 °C. Then, they were washed in TBST followed by incubation for 1 h at room temperature (RT) with corresponding secondary antibodies (HRP-goat anti-rabbit IgG or goat anti-mouse antibody, 1:10,000). The enhanced chemiluminescence (ECL) kit (Thermo Fisher Scientific) and the G: BOX Chemi X system (Syngene) were used for protein visualization.

### RNA interference

Small interfering RNAs (siRNAs) targeting GSDME were obtained from RiboBio (Guangzhou, China). Transfection of siRNA oligonucleotides was done via the Ribo FECT™ CP transfection kit (RiboBio, Guangzhou, China) as instructed by the manufacturer. Non‐targeted control siRNA (si-NC) was used as a negative control, and si-NC was also obtained from RiboBio (Guangzhou, China). After cell transfections, cells were incubated for 24‐48 h hours and were used as required. Lentivirus construction of GSDME knockdown was obtained from OBIO (Obio Technology Corp, China). shRNA-GSDME constructs targeting the GSDME cDNA sequence (5′-CAAGCAGCTGTTTATGACA-3′) were synthesized and cloned into the vectors. The SK-Hep1 cells were transfected with lentivirus (MOI = 20) in a 24-well plate. After transfection for 24 h, the fresh complete medium was then substituted, and the cells were cultured for an additional 72 h at 37 °C. The lentiviruses were used to stably knock down GSDME expression in SK-Hep1 cells, and the positive clones were selected in puromycin (5 ug/ml)-containing DMEM.

### Total RNA Extraction, Reverse Transcription, and qRT-PCR analysis

Total RNA of each cell line for RT-PCR analysis was collected in TRIzol (Takara, Japan). The RNA concentration was measured by NanoDrop 2000 (ThermoFisher Scientific). Then, 10 μg of total mRNA was used for cDNA synthesis utilizing a Vazyme HiScript Q RT SuperMix for qPCR (Vazyme, Nanjing, China) through S1000 Thermal Cycler (Bio-Rad, USA), as instructed by the manufacturer. qPCR was done on a 7900HT Fast Real-Time PCR System (ThermoFisher Scientific) with 2 × SYBR Green qPCR Master Mix (low ROX) kit (Bimake, China). All primers used for the qRT-PCR (BAK1, GSDME, NLRP6, and ACTB) were synthesized by TSINGKE (Beijing, China).

### Cell proliferation assay

A CCK-8 assay (MCE, HY-K0301, USA) was performed for the purpose of evaluating the proliferation level of cells in accordance with the manufacturer's protocol. Cells (2000/well) were inoculated in 96-well plates that contained 10 percent FBS-supplemented DMEM medium. Then, the medium was removed, and each well received 100 μl (10 μl CCK-8 + 90 μl medium) diluted CCK-8, followed by 1 h of cell incubation at a temperature of 37 °C. To assess HCC cell proliferation levels, absorbance at 450 nm was determined with the aid of a microplate reader (BioTek ELx800, USA).

### Colony formation assay

Transfected cells (500/well) were inoculated in 6-well plates that contained 10% FBS-supplemented DMEM medium and incubated for 10 days. Then, 4% paraformaldehyde was used to fix proliferating cell colonies stained with crystal violet (1%). Colonies with at least 50 cells were counted and imaged.

### Cell migration assay

A transwell chamber (3422, Corning, Costar, NY, USA) with an 8-micron pore size was employed in this assay. After transfection, SK-Hep1 cells (2 × 104 cells/well) were transferred to the upper chamber in a serum‐free medium. In the lower chamber, approximately 500 μl of media comprising 10% FBS was introduced. After a 24-h incubation period, the fixation of cells in paraformaldehyde (4 percent) and subsequent staining with crystal violet (1%) were carried out in the lower chamber. Carefully, cells that had not migrated to the lower chamber were removed. Then, 5 stochastic fields of view were microscopically imaged at × 20 magnification for analyses. Cell migration was also observed via the scratch wound (wound-healing) assay. Subsequently, the cells (5 × 105 cells/well) were inoculated in 6-well plates, subjected to incubation in 5% CO2 at 37 °C under conditions of saturated humidity overnight, and subsequently scraped a line by a 10 µl aseptic pipette tip. After incubation for 48 h, cells were washed thrice using PBS and microscopically imaged at 0 h and 48 h, respectively (SDPTOP, China). Wound healing rate was determined as follows: (average wound margin in 0 h—average wound margin in 48 h)/average wound margin in 0 h. Experiments were conducted in triplicates.

### In vivo study

Male BALB/c nude mice, 5-week-old, were obtained from Hunan Slake Jingda Laboratory Animal Co. Ltd. (Changsha, China). SK-Hep1 cells labeled with firefly luciferase (5 × 10^6^ cells in 200 µl PBS) were injected subcutaneously into the dorsal flank of nude mice. Twenty male BALB/c nude mice were assigned into two groups (sh-NC and sh-GSDME groups). The size of the xenograft tumors was measured every five days for a month and calculated as follows: V = 0.5 × L × W^2^ (V, volume; L, length; W, width). The mice were maintained according to the criteria of the National Institutes of Health animal use guidelines. All experimental procedures were approved by the Ethics Committee of Tongji Hospital Institutional Review Board.

### Live animal imaging

The Lago X in vivo imaging system (Spectral Instruments Imaging) was used for the detection of luciferase activity in living animals. The mice were anesthetized with pentobarbital sodium (1.5%, 50 mg/kg), intraperitoneally administered 150 mg/kg of D-luciferin, and images were captured 10 min after injection with the Lago X.

### Statistical analyses

The Wilcox rank-sum test function in the R software was used for the purpose of determining the PRGs having remarkably different levels of expression between tumor samples and normal controls. The Chi-square test was applied in the comparison of the distribution of the clinical-pathological parameters between high- and low-risk groups in LIHC. The Kaplan–Meier curves were examined by performing the log-rank test, which was employed to contrast the overall survival (OS) across various groups. The cutoff for significance was set as *p* < 0.05. The R studio (version: 4.0.2) was utilized to conduct all of the analyses in the present research. Some statistical analyses were performed using GraphPad Prism 9 software (GraphPad Software, Inc.), and quantitative data are presented as the mean ± standard error of the mean (SEM) from three independent experiments. Statistical differences between the two groups were compared using a paired Student's t-test, whereas comparisons between > 2 groups were based on one-way ANOVA. *p* < 0.05 was considered to indicate a statistically significant difference.

## Results

### Prognostic pyroptosis-associated DEGs

**Fig. 1:**
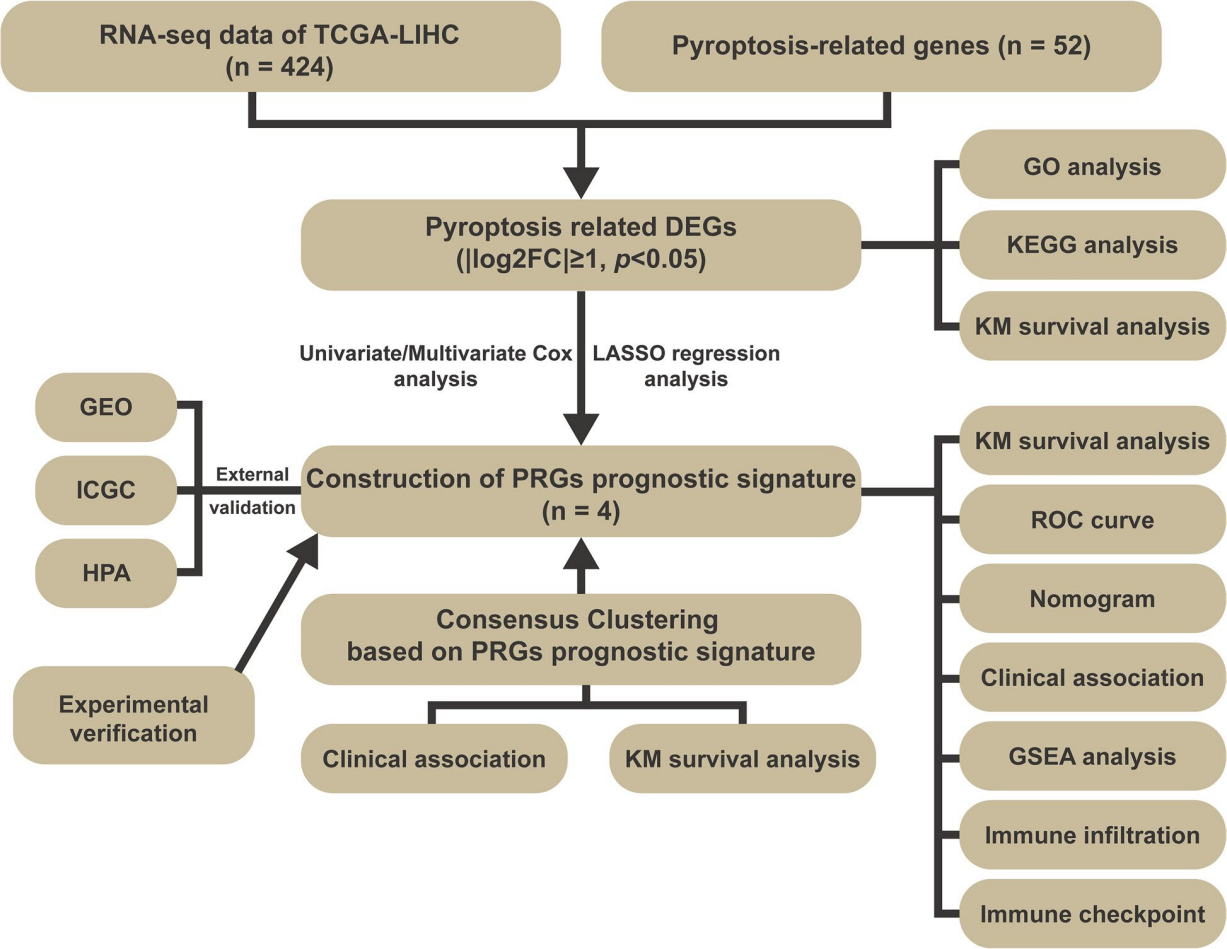
The flowchart of this study

**Fig. 2:**
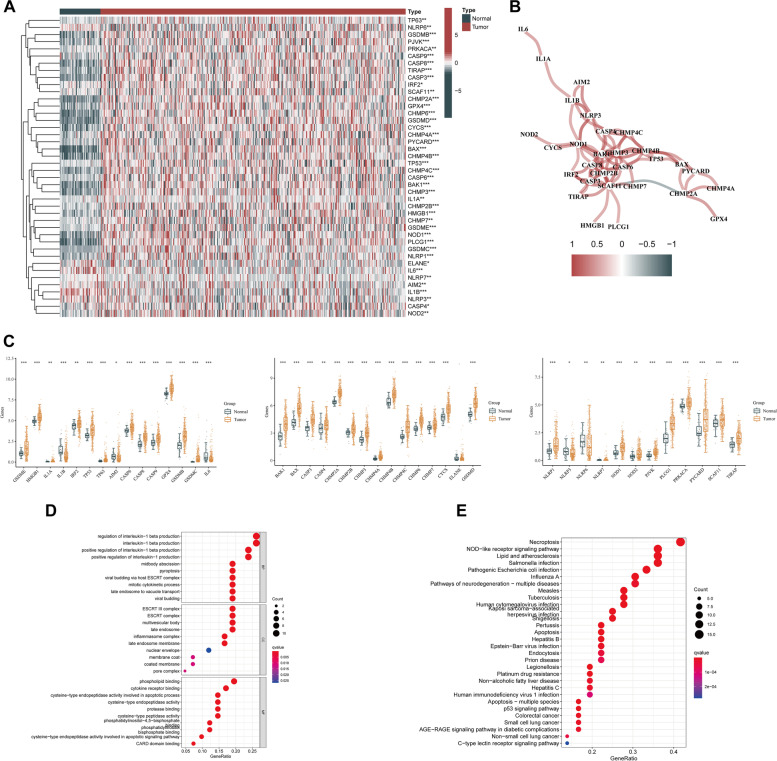
Differential expressions of PRGs and their interaction. **A**, **C** The heatmap and histogram of the differentially expressed PRGs between LIHC tissues and non-cancerous tissues. **B** The correlation network of the differentially expressed PRGs. Red and blue lines indicate positive and negative correlations, respectively. The intensity of the color represents the strength of the relevance. **D**, **E** GO and KEGG pathway enrichment of the differentially expressed PRGs

The flow diagram of the current study is shown in Fig. 1. Forty-two PRGs were established as having differential expression between tumor and the adjoining non-tumor samples. DEGs in the two groups are presented in the form of heatmap and histogram (*p* < 0.05; Fig. 2A, C). Associations among the differentially expressed PRGs are shown in Fig. 2B. To evaluate the underlying molecular purpose as well as potential signaling pathways associated with PRGs, GO and KEGG analyses were done. GO analysis demonstrated a remarkable enrichment of PRGs in modulating interleukin − 1 beta production, interleukin − 1 beta production, and positively modulating interleukin − 1 beta production in BP. CC was highly up-modulated in ESCRT III complex, ESCRT complex, and multivesicular body. MF mainly modulated cytokine receptor binding, phospholipid binding, and cysteine − type endopeptidase activity implicated in apoptosis (Fig. 2D). KEGG analyses illustrated a remarkable enrichment of 42 PRGs in necroptosis, NOD-like receptor signaling pathway, lipid and atherosclerosis, pathogenic *Escherichia coli* infection, salmonella infection, and influenza A (Fig. 2E).

### Establishment of pyroptosis-related genes prognostic signature

**Fig. 3:**
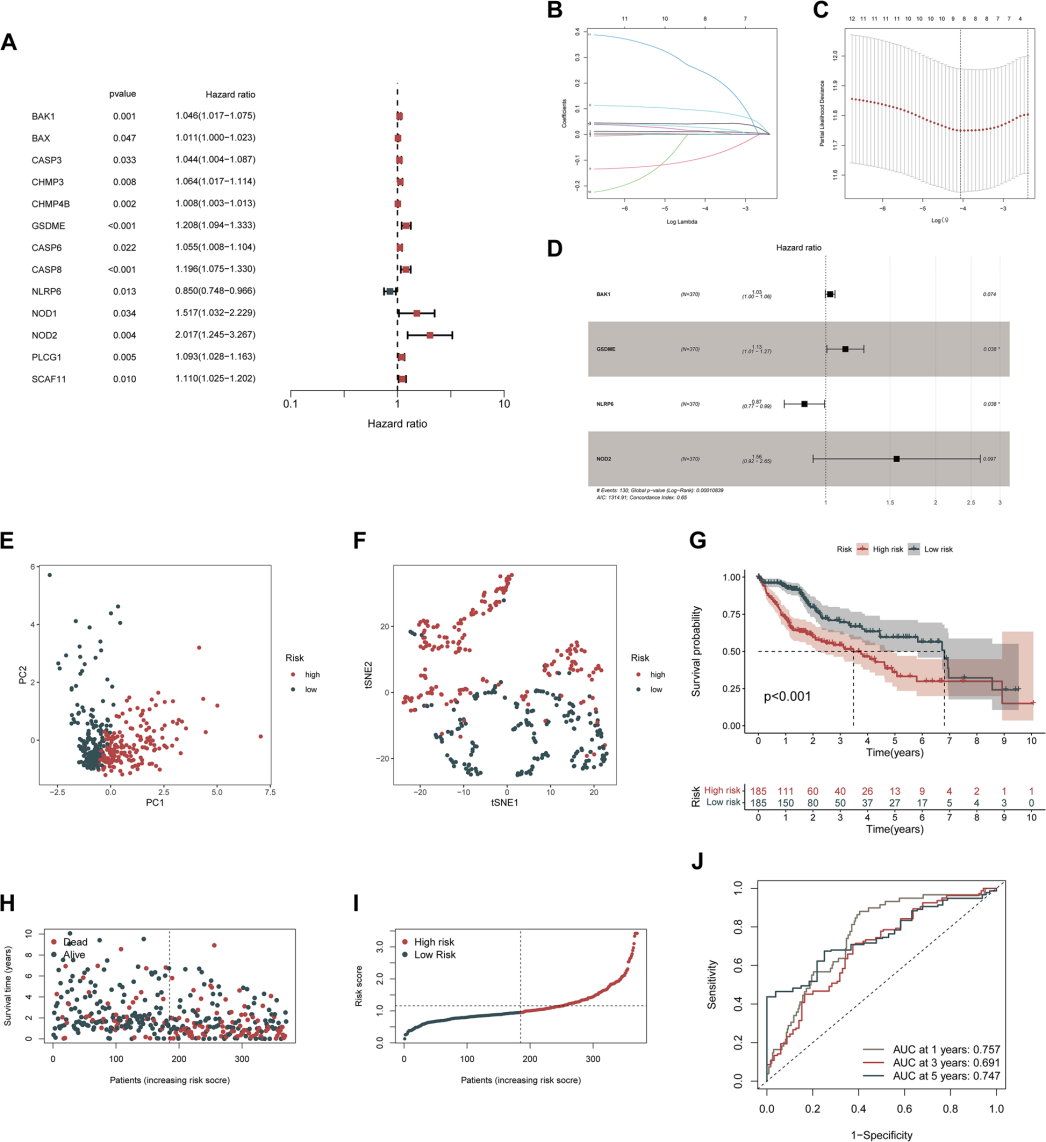
Construction of PRGs prognostic signature for LIHC. **A** Forrest plot of prognostic PRGs identified by univariate Cox regression analysis. PRGs with *p* < 0.05 were shown. **B**, **C** The Lasso regression analysis identified 8 PRGs and the optimal penalty parameter λ was obtained through ten-fold cross-validation. **D** Forest plot of multivariate Cox proportional hazards regression model presenting the HRs for the PRGs prognosis model containing 4 PRGs. **E**, **F** PCA and tSNE plot for LIHC based on the risk score. **G** Kaplan–Meier survival curve of the OS for LIHC patients in the high-/low-risk group. **H** Distribution of risk score based on the prognostic model. **I** The scatter plot of all patients’ survival status. **J** ROC curves for the predicting OS at 1, 3, and 5 years. ROC, receiver operating characteristic curves. *p < 0.05

The univariate Cox regression analyses were performed to screen PRGs with prognostic significance, and 13 PRGs that were associated with OS in the TCGA-LIHC cohort were identified (BAK1, BAX, CASP3, CHMP3, CHMP4B, GSDME, CASP6, CASP8, NLRP6, NOD1, NOD2, PLCG1, SCAF11) (*p* < 0.05) and details of them are depicted by the forest plot (Fig. 3A). All 13 PRGs, apart from NLRP6, were defined as risk genes with HRs > 1. Kaplan–Meier survival analysis of the PRGs screened out by univariate Cox analysis is shown in Supplementary Figure S1 (*p* < 0.05). The Lasso regression method was employed to prevent the model from overfitting, and eight genes were screened with the optimum penalty value (λ) selected by ten-fold cross-validation (Fig. 3B, C). Subsequently, according to the findings of multivariate Cox regression, four PRGs, including BAK1, GSDME, NLRP6, and NOD2, were incorporated into the PRGs prognostic signature and were determined as critical biomarkers and independent prognostic markers (Table 1; Fig. 3D). Each patient’s risk score was determined according to the coefficient of four PRGs in prognostic signature using the above-mentioned formula: the risk score = (0.028) * BAK1 + (0.124) * GSDME + (− 0.135) * NLRP6 + (0.447) * NOD2. Afterward, after setting the median risk score as the threshold criterion, patients in the LIHC cohort were classified into high- (*n* = 185) and low-risk (*n* = 185) groups. PCA revealed efficient discernment of patients with various risks into two subgroups (Fig. 3E). Furthermore, the PCA result was validated by t-distributed stochastic neighbor embedding (tSNE) (Fig. 3F). The Kaplan–Meier survival curves revealed that patients belonging to the high-risk group had a much lower chance of survival compared to individuals belonging to the low-risk group (*p* < 0.001) (Fig. 3G). Carrying out a comparative analysis between the risk score and the matching distribution of survival status, high-risk group patients exhibited higher risk scores and corresponding higher mortality rates relative to the low-risk group (Fig. 3H, I). In addition, we employed time-dependent ROC curves to investigate the performance of the risk prediction model. Respectively, the AUCs of the model for the four PRGs were 0.757, 0.691, and 0.747 at 1, 3, and 5 years (Fig. 3J). Collectively, based on these findings, it is reasonable to hypothesize that the risk score model integrating these four independent prognostic markers was capable of accurately predicting the prognosis status of patients with LIHC.

**Table 1:** Multivariate Cox proportional hazards regression model including 4 pyroptosis-related genes as the independent prognostic biomarkers for overall survival in patients with LIHC

pyroptosis-related genes	Coefficient	HR	95%LowerCI	95%UpperCI	*p*-value
BAK1	0.028	1.028	0.997	1.060	0.074
GSDME	0.124	1.131	1.007	1.271	0.038 *
NLRP6	-0.135	0.874	0.770	0.993	0.038 *
NOD2	0.447	1.563	0.923	2.648	0.097

*HR* Hazard ratio, *CI* Confidence interval

^*^p < 0.05

### Hierarchical analysis of predictive performance in the pyroptosis-associated genes prognostic signature based on clinicopathological characteristics

**Fig. 4:**
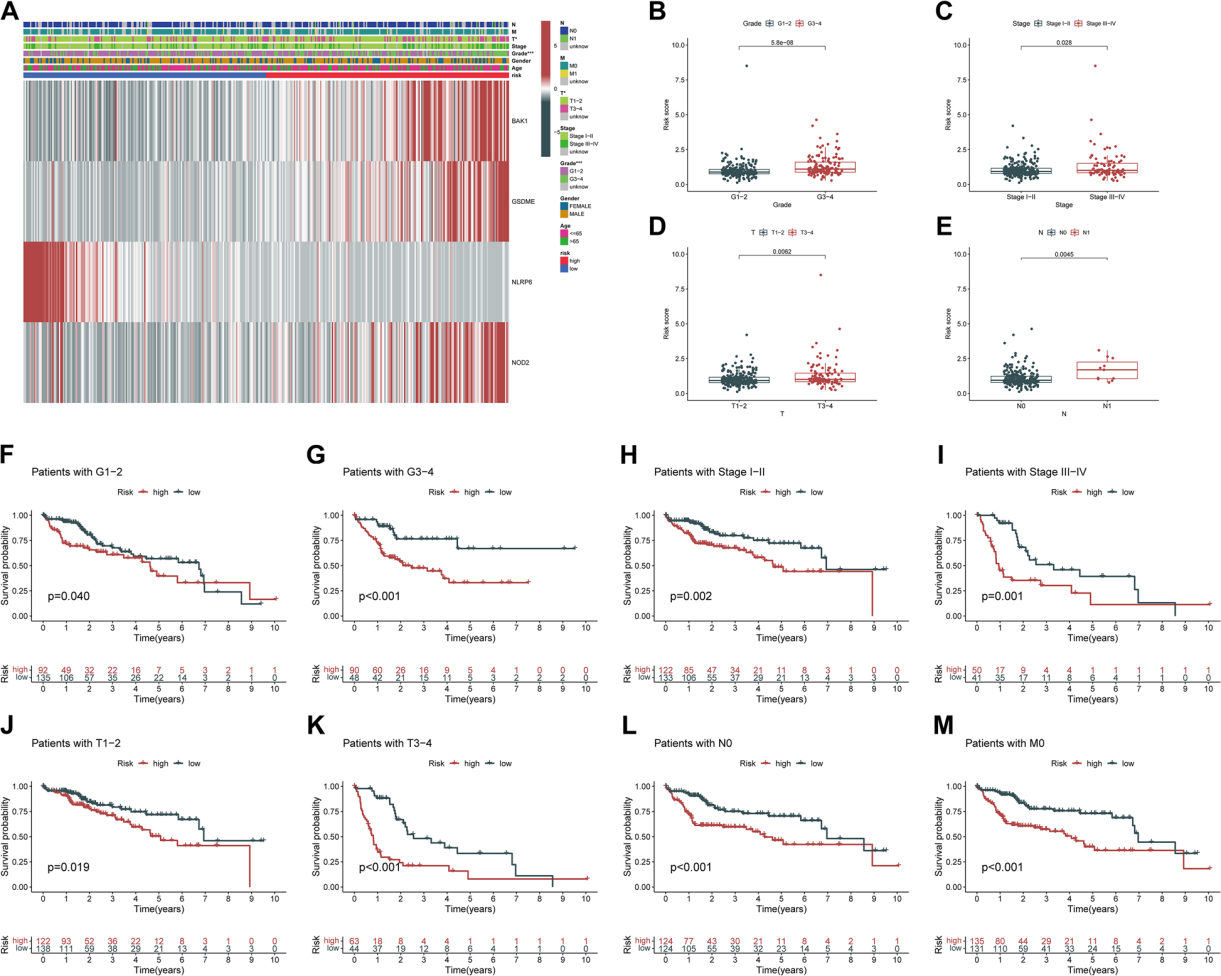
The associations between the risk score and different clinicopathological features. **A** Heatmap for graphically illustrating the connections between clinicopathological features and the expression levels of the 4 PRGs from risk model. **B-E** Clinicopathological characteristics (Grade, Stage, T-staging and N-staging) of patients with different risk scores. Chi-square test was applied to determine whether the correlations between clinical features and risk score were statistical significance. **F-M** Kaplan–Meier curves of OS between each two groups stratified by different clinicopathological features. *p < 0.05, ***p < 0.001

It is widely known that clinicopathological characteristics such as grade, stage, TNM-staging, age, and gender were considered relevant factors to overall survival. To thoroughly comprehend the models’ prognostic values, we subsequently validated whether there existed a correlation between clinicopathological features and the risk score model incorporating four PRGs. Hence, the Chi-Square test was applied for determining whether the PRGs prognostic signature had a sort of predictive effectiveness for anticipating the clinical and pathological variables in patients with LIHC. The heatmap was employed to visualize the findings of the Chi-Square (Fig. 4A), which illustrated marked discrepancies in the distribution of T-staging (*p* < 0.01) and Grade (*p* < 0.001) between low- and high-risk groups. The heatmap demonstrated that the expression levels of NLRP6 exhibited negative correlations with the risk score and could be used as unfavorable prognostic markers in an independent manner, whereas the expression levels of the BAK1, GSDME, and NOD2 showed positive correlations and served as unfavorable prognostic markers (Fig. 4A). We additionally conducted stratification analyses in order to evaluate whether the PRGs prognostic signature maintained its prediction potency across multiple subgroups. The findings illustrated that there were indeed some distinct differences in risk scores across different subgroups in T-staging, N-staging, Stage, and Grade (*p* < 0.05). Moreover, greater risk scores were associated with more advanced clinical-pathological characteristics (Fig. 4B-E), but neither Age nor Gender was significantly correlated with the risk scores, and the number of subjects in M1 was too few for statistical analysis (not presented in Figure). The Kaplan–Meier survival analysis suggested that only the patients with N1 and M1 resulted in no remarkable difference (*p* > 0.05; the reason for this might be that the number of patients was inadequate to make significant comparisons, not shown in Figure), apart from these subgroups, patients with higher risk exhibited unfavorable OS status in all of the Stage, Grade, T-staging, N0 and M0 subgroups (Fig. 4F-M). In general, these results imply that the PRGs prognostic signature is a viable predictor for patients with LIHC.

### Establishment of nomogram generated from PRGs prognostic signature

**Fig. 5:**
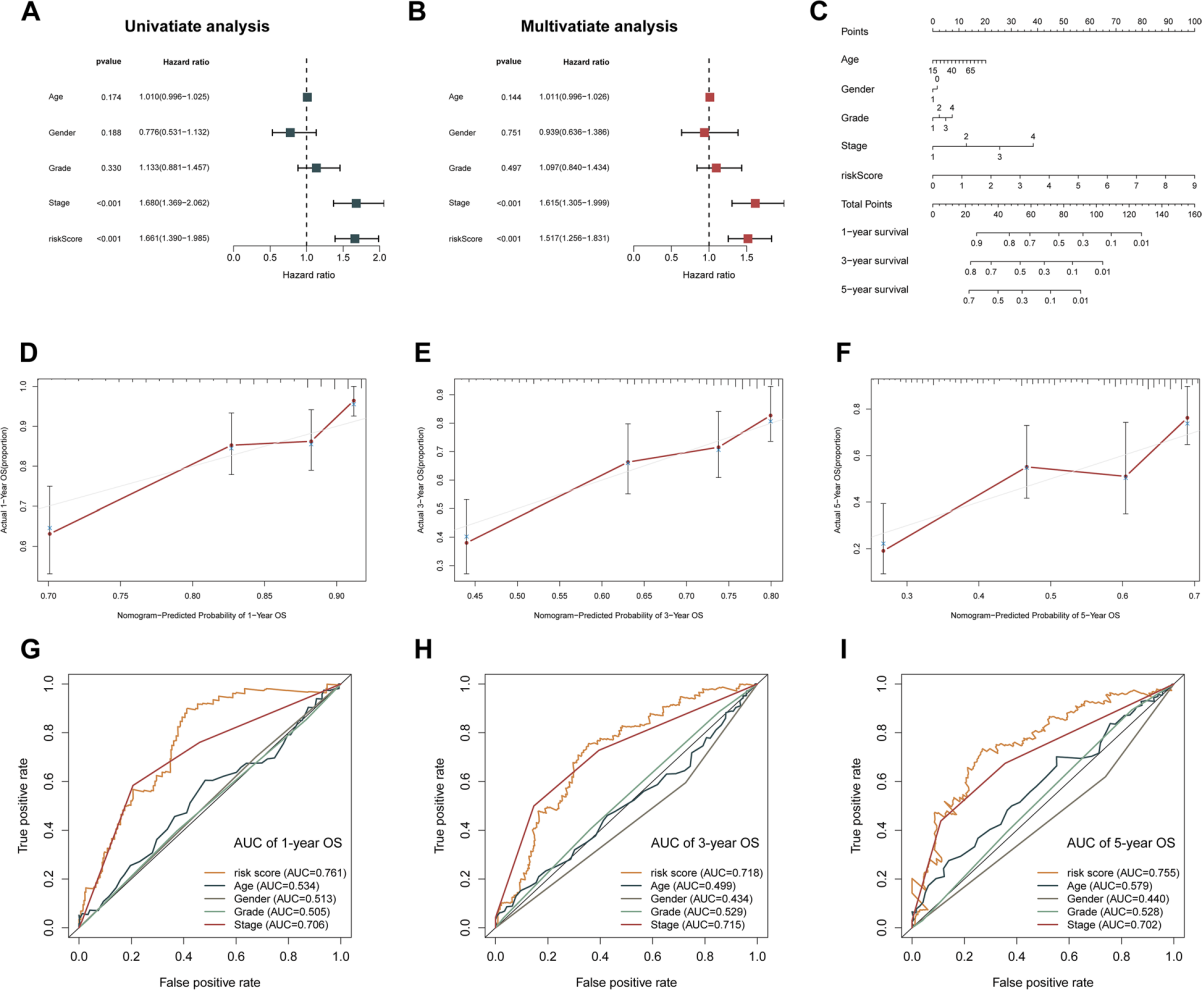
Evaluation of PRGs prognostic signature and nomogram construction in LIHC. **A**, **B** Univariate and multivariate Cox regression analyses according to risk score and clinical characteristics in LIHC patients. **C** A nomogram integrated risk score derived from the PRGs prognostic signature and clinicopathological features. **D-F** Calibration curve of the nomogram showed a good predictive ability for predicting 1-, 3-, and 5-years OS in LIHC patients. **G-I** AUC of ROC curves assessed the prognostic accuracy for predicting 1-, 3-, and 5-years OS in LIHC patients

According to the results of the univariate Cox analysis, the PRGs prognostic signature served as an adverse prognostic indicator for OS (Hazard ratio: 1.661, 95% CI: 1.390 − 1.985, *p* < 0.001; Fig. 5A). Then, by performing the multivariate Cox analysis, we demonstrated that the prognostic signature acted as a predictor of unfavorable prognosis status in an independent manner, which further supported our findings (Hazard ratio: 1.517, 95% CI: 1.256 − 1.831, *p* < 0.001; Fig. 5B). Based on these findings, we hypothesized that this prognostic signature can function as a prognostic marker in an independent manner and that it may have pragmatic use in clinical prognostic evaluation. A nomogram consisting of clinical-pathological parameters, including stage, grade, gender, age, and the newly created riskScore, was designed for the purpose of developing a clinically-applicable quantifiable method for estimating the survival chances of patients with LIHC (Fig. 5C). As indicated by the red line (the true observation), which provides a positive fit of the grey line (the reference line), the calibration plots for the prognostication of survival chances over 1, 3, and 5 years revealed remarkable consistencies between the nomogram-predicted outcomes and the observed results (Fig. 5D-F). Moreover, the ROC curve had a satisfactory accuracy and discriminating ability of the PRGs prognostic signature with AUC values of 0.761, 0.718, 0.755, for 1-, 3- and 5-year OS, correspondingly (Fig. 5G-I). Therefore, the PRGs signature was established to be accurate and suitable for prognostic prediction of LIHC.

### Consensus clustering of PRGs prognostic signature recognized two clusters of LIHC patients with remarkably distinct prognosis status

**Fig. 6:**
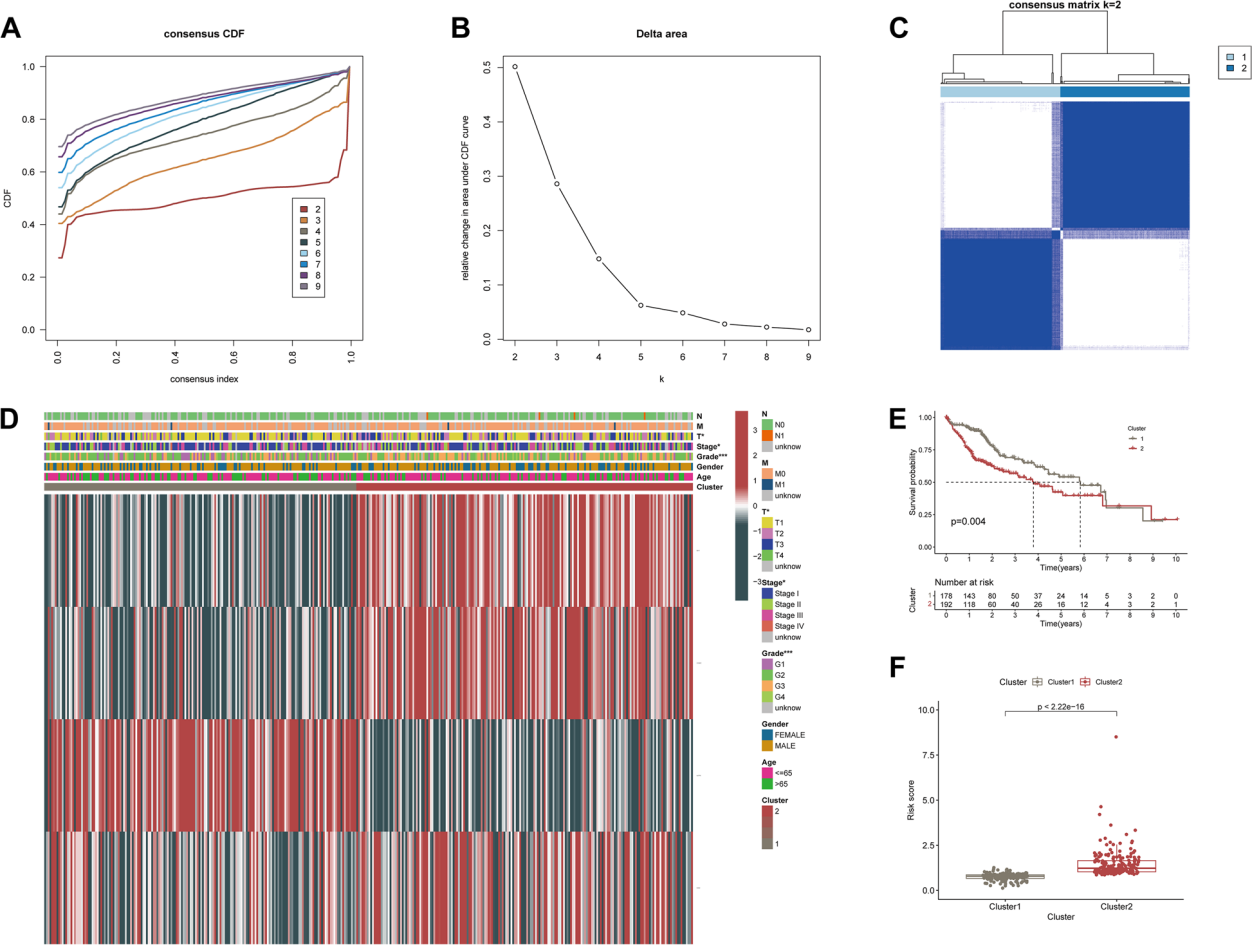
Differential clinicopathological features and survival of LIHC in two clusters. **A** Consensus clustering cumulative distribution function (CDF) for k = 2–10. **B** Relative change in area under CDF curve for k = 2 to k = 10. **C** Consensus clustering matrix for k = 2. **D** Heatmap and the clinicopathological characters of the two clusters classified by PRGs prognostic signature. **E** Kaplan–Meier curves of OS for patients with LIHC in the two clusters. **F** Boxplot of risk score derived from the PRGs prognostic signature in the two clusters

We clustered a total of 370 LIHC patients into two distinct molecular subgroups through the R package “ConsensusClusterPlus”, designated as cluster1 (*n* = 178) and cluster2 (*n* = 192) according to the expression patterns of four PRGs in the prognostic signature aforementioned (Table 1). With the aid of the ConsensusClusterPlus R software, it was determined that k = 2 was the best option for the minimum crossover among LIHC samples after evaluating a range of values from 2 to 10 in the LIHC cohort (Fig. 6A-C). As a result, we divided LIHC patients into two clusters, named cluster1 and cluster2. We subsequently examined the clinical-pathological parameters between these two subgroups, and Chi-square test findings illustrated that the two LIHC clusters were remarkably associated with Stage (*p < 0.05), Grade (***p < 0.001), and T-staging (*p < 0.05) (Fig. 6D), which suggested that these clinical variables exhibited significantly varied ratio between the two LIHC clusters. The Kaplan–Meier curves illustrated a prolonged OS duration among patients from cluster1 unlike the case among the patients from cluster2 (log-rank test, *p* = 0.004; Fig. 6E), which showed strong consistency with the findings that cluster2 exhibited a higher level of PRGs prognostic signature-based risk score as opposed to cluster1, as presented in Fig. 6F (*p* < 0.001). These findings offered additional proof to support the conclusion that the prognostic signature of PRGs was significantly correlated with the prognosis of patients with LIHC.

### Analysis of Gene Set Enrichment Analysis (GSEA)

**Fig. 7:**
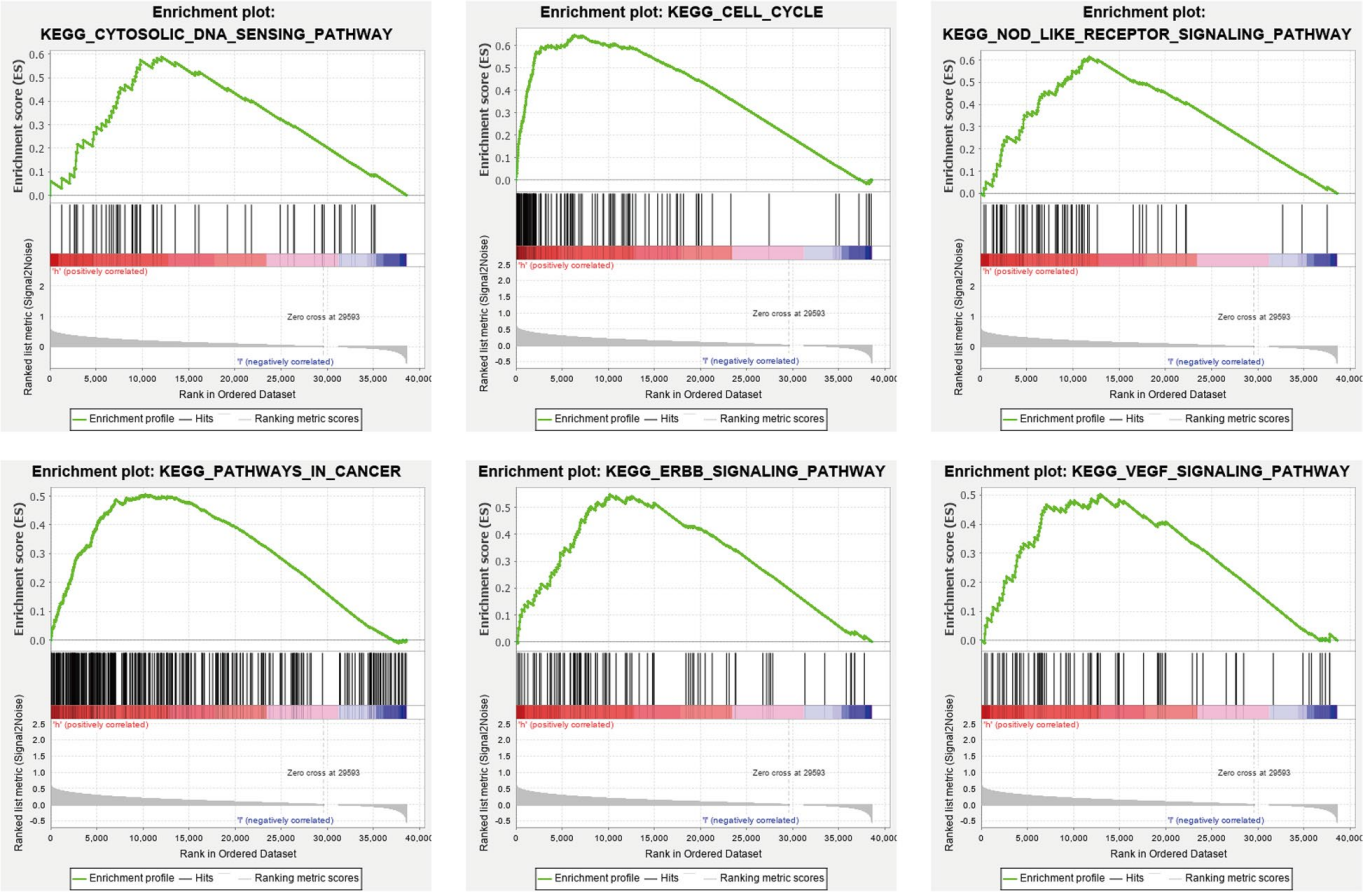
Gene set enrichment analysis. significant level was NOM p-val < 0.05 and | NES |> 1

Given the possible role of pyroptosis during the tumorigenesis and the interesting results we found above, we evaluated the role of PRGs prognostic signature in the pathogenesis of LIHC. GSEA was utilized for the purpose of examining the possible mechanisms of the PRGs prognostic signature involved in LIHC progression. The results indicated that cytosolic DNA-sensing pathway, cell cycle, NOD-like receptor signaling pathway, pathways in cancer, ErbB signaling pathway, and VEGF signaling pathway were activated by the high-risk group of the PRGs prognostic signature (NOM p-val < 0.05 and | NES |> 1; Fig. 7). These results imply that high risk was highly associated with pyroptosis, tumor growth as well as development.

### External validation of PRGs prognostic model

**Fig. 8:**
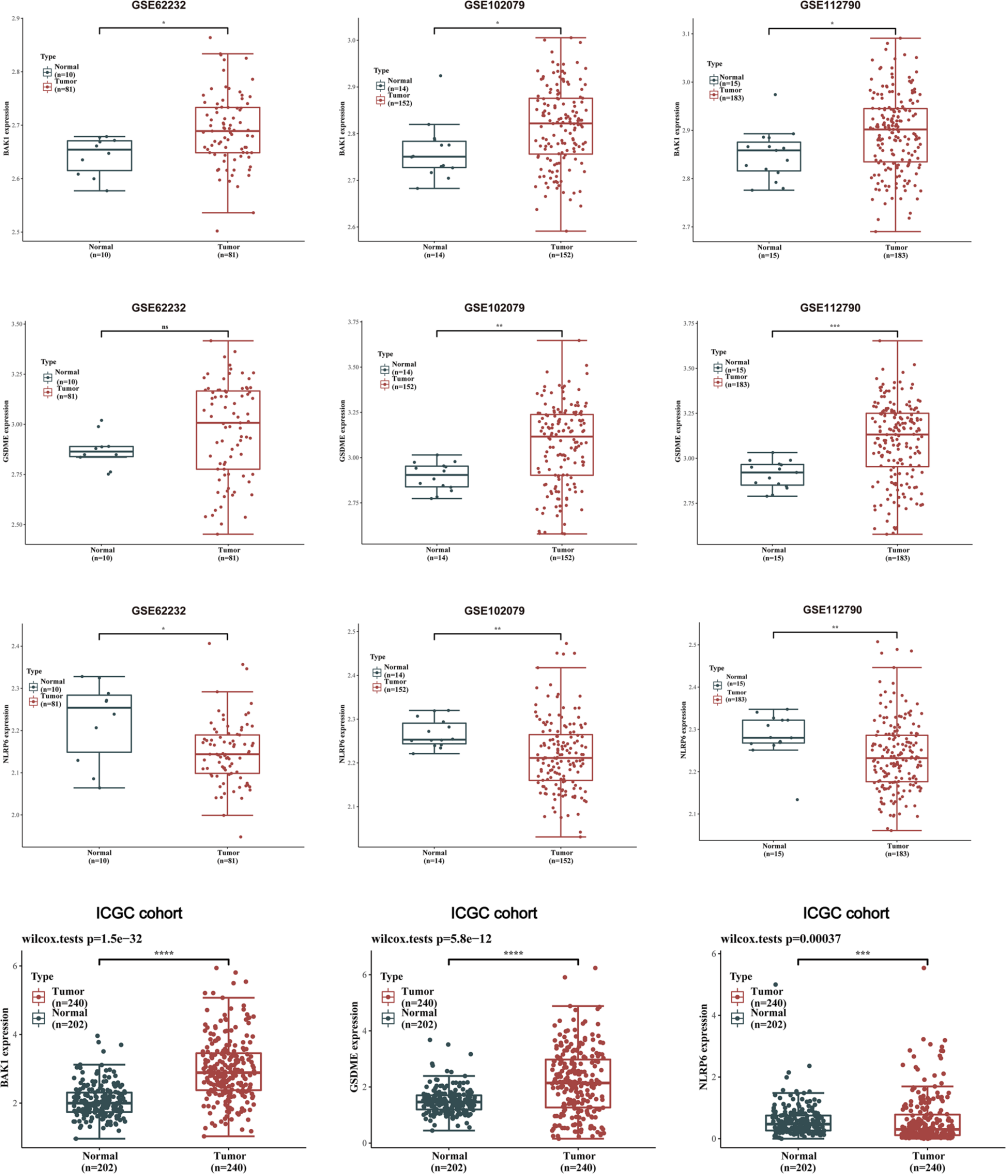
External validation of the prognostic model in the GEO and ICGC cohorts. Box plot indicating higher expression of BAK1 and GSDME in HCC tissues than those in normal liver tissues and the opposite trend of NLRP6. *p < 0.05, **p < 0.01, ***p < 0.001, ****p < 0.0001

**Fig. 9:**
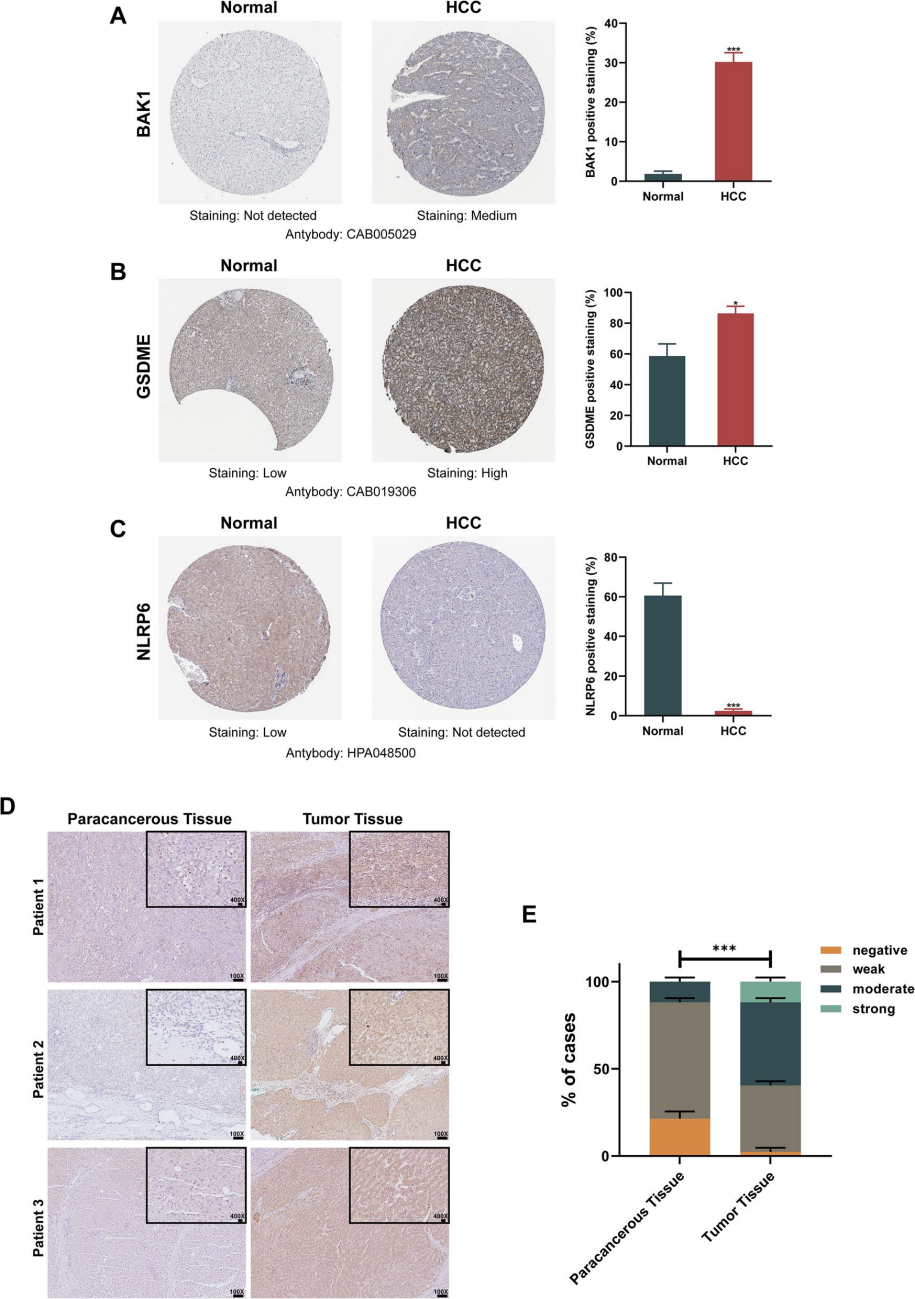
Representative immunohistochemistry images of the PRGs between HCC tissues and normal liver tissues. **A-C** The protein expression data of BAK1, GSDME and NLRP6 were retrieved from the HPA database (the Human Protein Atlas). IHC quantification is shown on the right, respectively. **D** Immunohistochemical staining of GSDME protein expression in 14 pairs of clinical HCC and corresponding paracancerous tissues. Scale bars: 100X = 100 μm; 400X = 20 μm. **E** Pathological appraisal of GSDME expression in between HCC and adjacent noncancerous tissues. Data analyzed by two-way ANOVA. ***p < 0.001

To further validate the value of three of the PRGs in the prognostic model (BAK1, GSDME, and NLRP6) for the development of the diagnostic as well as prognostic signatures, we used HCC cohorts from the GEO database (GSE62232, GSE102079, and GSE112790) and ICGC database to assess the expressions of the three PRGs. As shown in Fig. 8, the results of additional independent validation in GSE62232, GSE102079, and GSE112790 as well as in the ICGC cohort are consistent with the expression trend of three PRGs in our results. Compared with normal tissues, BAK1 and GSDME levels were markedly elevated in HCC tumor tissues, meanwhile, the expression of the NLRP6 showed the opposite trend as decreasing in HCC tumor samples in contrast with normal samples. Immunohistochemistry (IHC) staining revealed that protein levels of BAK1 and GSDME in HCC samples were elevated in contrast with normal samples and the protein expression of the NLRP6 showed the opposite trend, in tandem with their mRNA expression tendency (Fig. 9A-C). Further assessment of GSDME protein expression was conducted by IHC staining on HCC samples and corresponding paracancerous tissues. The results revealed that most HCC samples exhibited higher protein expression of GSDME compared to corresponding paracancerous tissues, and quantification indicated statistical significance (Fig. 9D, E).

**Fig. 10:**
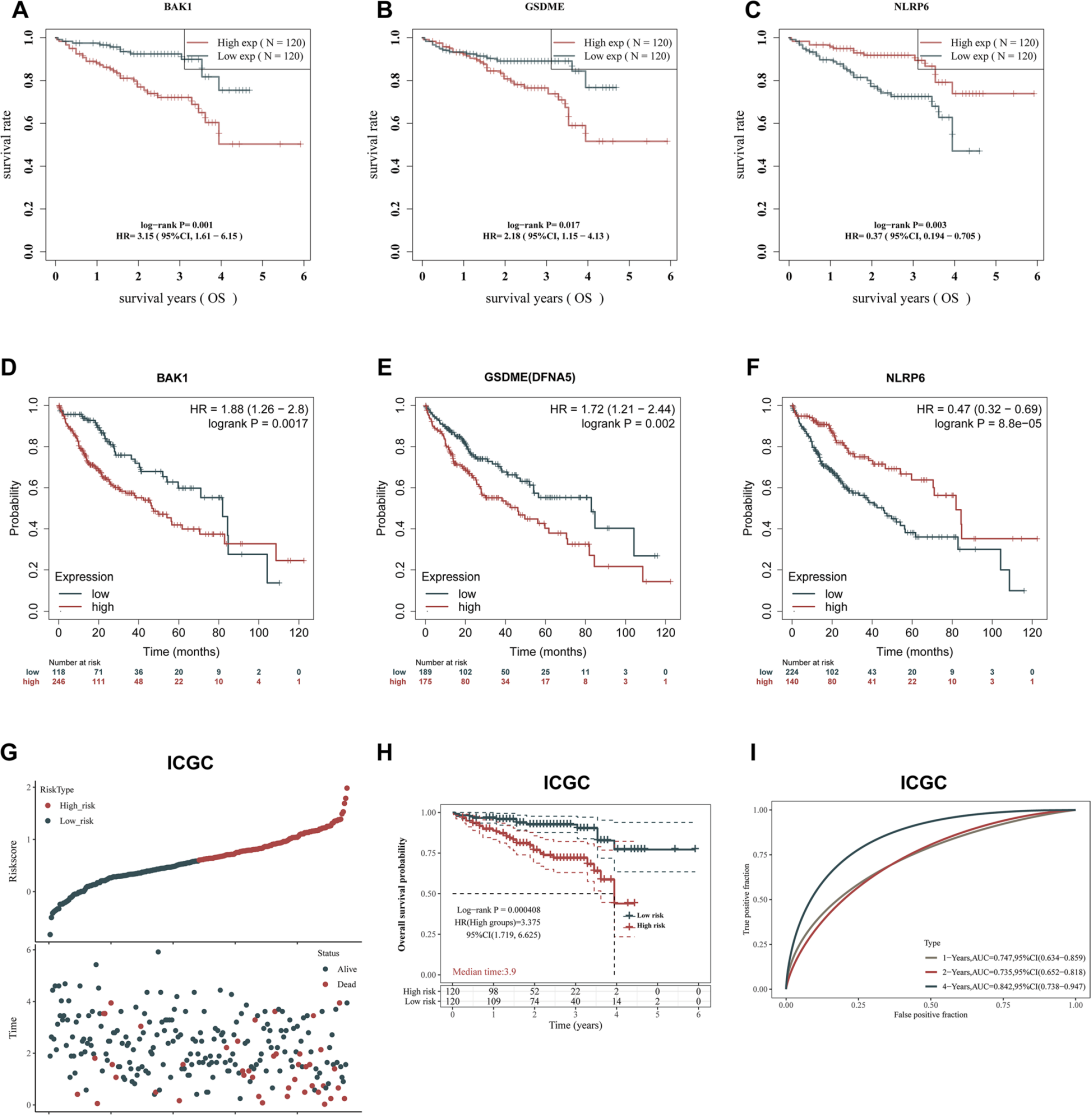
Validation of the prognostic values of hub PRGs in LIHC patients. **A-F** OS analysis of hub PRGs in ICGC (**A-C**) and Kaplan–Meier plotter (**D-F**). **G** Distribution of risk score based on the prognostic model (upper panel). The scatter plot of all patient’s survival status (lower panel). **H** Kaplan Meier survival curve of OS for high and low risk groups in ICGC cohort. **I** ROC curves for the predicting OS at 1, 2 and 4 years. Hazard ratio (HR) and log-rank P values are shown

ICGC database and Kaplan–Meier Plotter revealed that the high expressions of BAK1 and GSDME markedly decreased the survival rate of LIHC patients. On the contrary, LIHC patients with elevated NLRP6 levels exhibited longer survival in comparison with the patients with suppressed NLRP6 levels (*p* < 0.01, Fig. 10A-F). Furthermore, validation in ICGC cohort as external set confirmed prognostic value of the PRGs prognostic signature. To fully show the applicability of the PRGs prognostic signature in this independent external dataset, risk scores distribution, survival status of each patient, KM curve and ROC curve were conducted (Fig. 10G-I). These external validation results further verified the independent prognostic values of the PRGs prognostic signature in LIHC patients.

### Tumoral effects of pyroptosis regulator GSDME in HCC cells

**Fig. 11:**
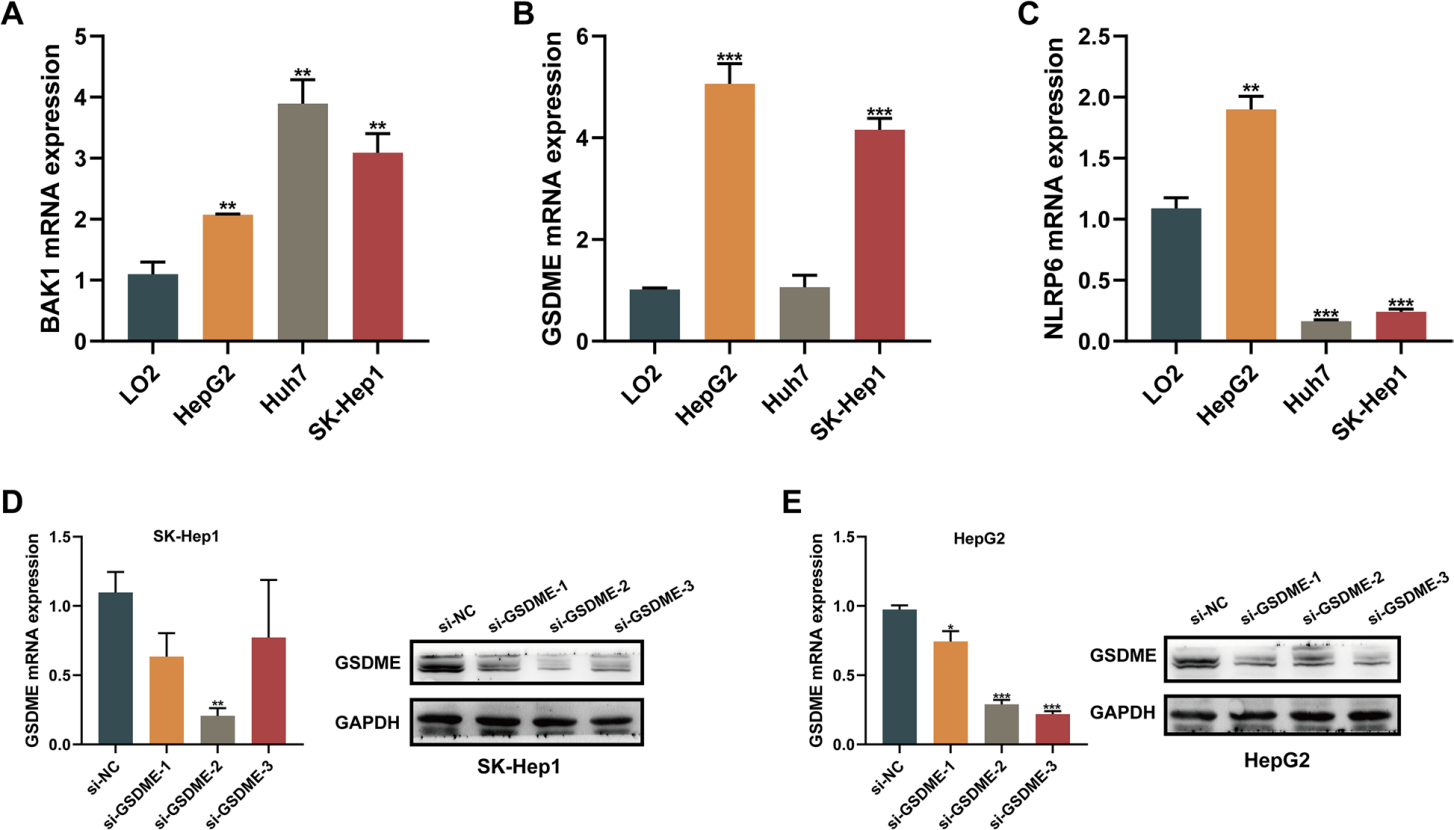
Validation of GSDME-targeting siRNA transfection efficiency. **A-C** qPCR was used to measure the mRNA level of BAK1 (**A**), GSDME (**B**) and NLRP6 (**C**) in human LO2 hepatocytes and HCC cell lines. **D**, **E** Silencing of GSDME expression in SK-Hep1 (**D**) and HepG2 (**E**) cells using siRNAs. RT-PCR and western blot analyses showed that GSDME-targeting siRNA si-GSDME-2 provided optimal depletion of GSDME compared to the siRNA-negative control (si-NC) in both cell lines. The data were expressed as means ± SEM. *p < 0.05, **p < 0.01, ***p < 0.001

**Fig. 12:**
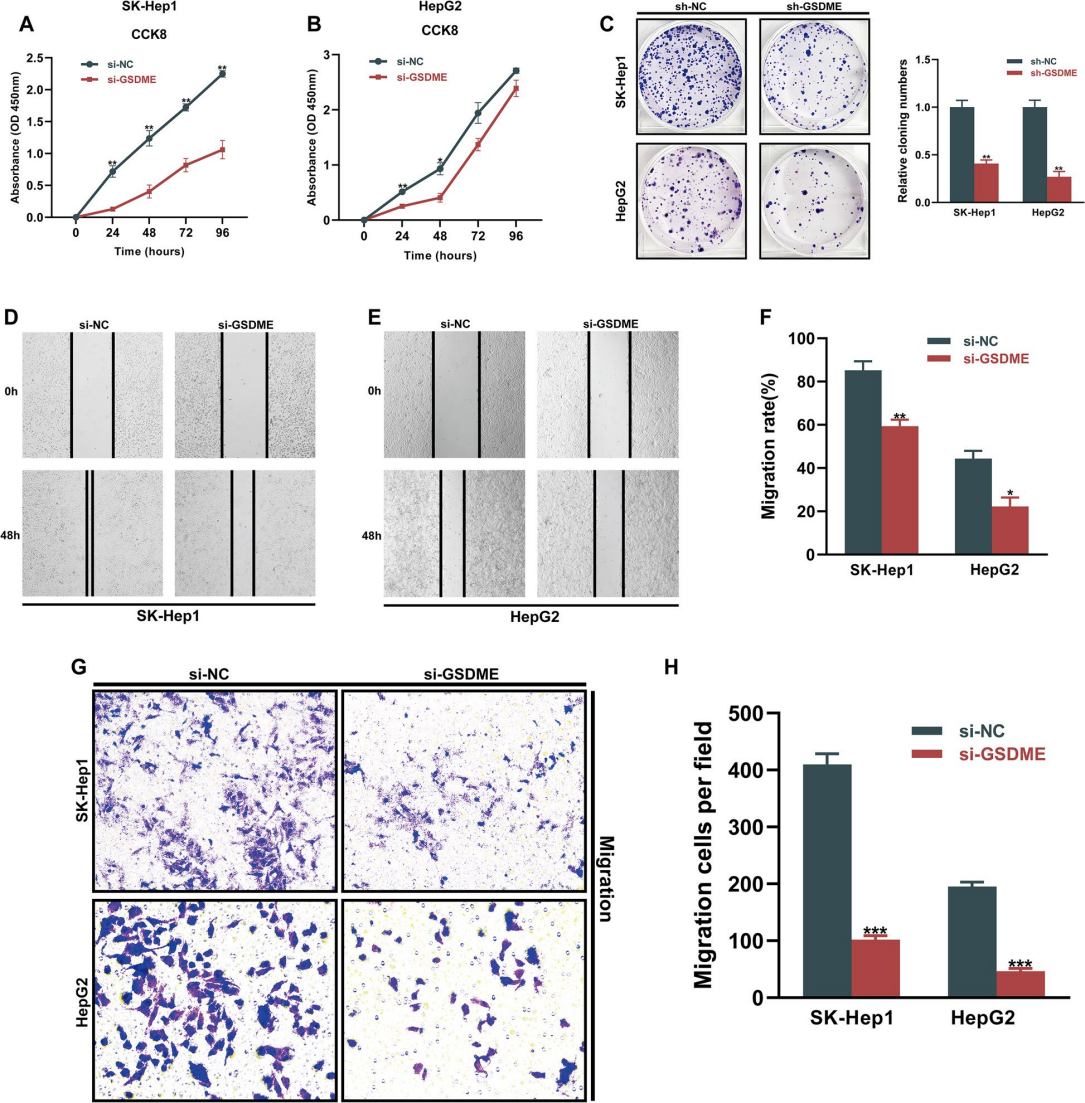
Effects of GSDME silencing on proliferation and migration in HCC cells. **A**, **B** CCK‐8 assay indicated that GSDME-knockdown significantly decreased the proliferation rate of HCC cells. **C** Change of colony formation ability of HCC cells treated with sh-NC and sh-GSDME. **D-F** Scratch assay of HCC cells treated with si-NC and si-GSDME. **G**, **H** Transwell assays were performed with si-NC and si-GSDME-transfected HCC cells to determine the effects of GSDME on cell migration. The bar graphs (**C**, **F**, **H**) show the results of quantitative analyses. The data were expressed as means ± SEM. *p < 0.05, **p < 0.01, ***p < 0.001

In this assay, mRNA levels of BAK1, GSDME, and NLRP6 in various cell lines were determined. BAK1 and GSDME levels in HCC cells were established to be elevated, relative to human LO2 hepatocytes (Fig. 11A-C). Based on qPCR results, we selected SK-Hep1 and HepG2 cell lines for subsequent analysis, which showed a relatively consistent trend with our results from bioinformatics analysis. To assess the oncogenic roles of critical pyroptosis regulators in HCC cells, we selected GSDME, the member of the gasdermin superfamily, for subsequent experiments since GSDME showed the most prominent up-regulation in both mRNA and protein levels ((Figs. 9B and 11B), and pyroptosis can be viewed as a gasdermin-mediated programmed necrosis since the gasdermin family plays an indispensable role in pyroptosis [6]. Thus, we silenced GSDME in SK-Hep1 and HepG2 cells. The efficiency of siRNA was verified via qPCR and Western blot. GSDME expression was obviously inhibited with GSDME siRNA administration and si-GSDME-2 exhibited the highest knockdown efficiency (> 70%) at both protein and mRNA levels in both cell lines (Fig. 11D, E), thus si-GSDME-2 was used in subsequent assays. The CCK8 analysis revealed a significant reduction in HCC cells proliferation following GSDME-knockdown (*p < 0.05, **p < 0.01, Fig. 12A, B). Furthermore, the results of the colony formation assay provided supporting evidence of the effect of GSDME on HCC cells proliferation (Fig. 12C). The results from wound healing as well as transwell migration assays indicated that knockdown of GSDME partially blocked the migration in HCC cells (Fig. 12D-H). These findings revealed that proliferation, as well as migration of SK-Hep1 and HepG2 cells, were decreased while silencing GSDME expression.

### Knocking down GSDME reduces the growth of HCC cell xenografts in Vivo

**Fig. 13:**
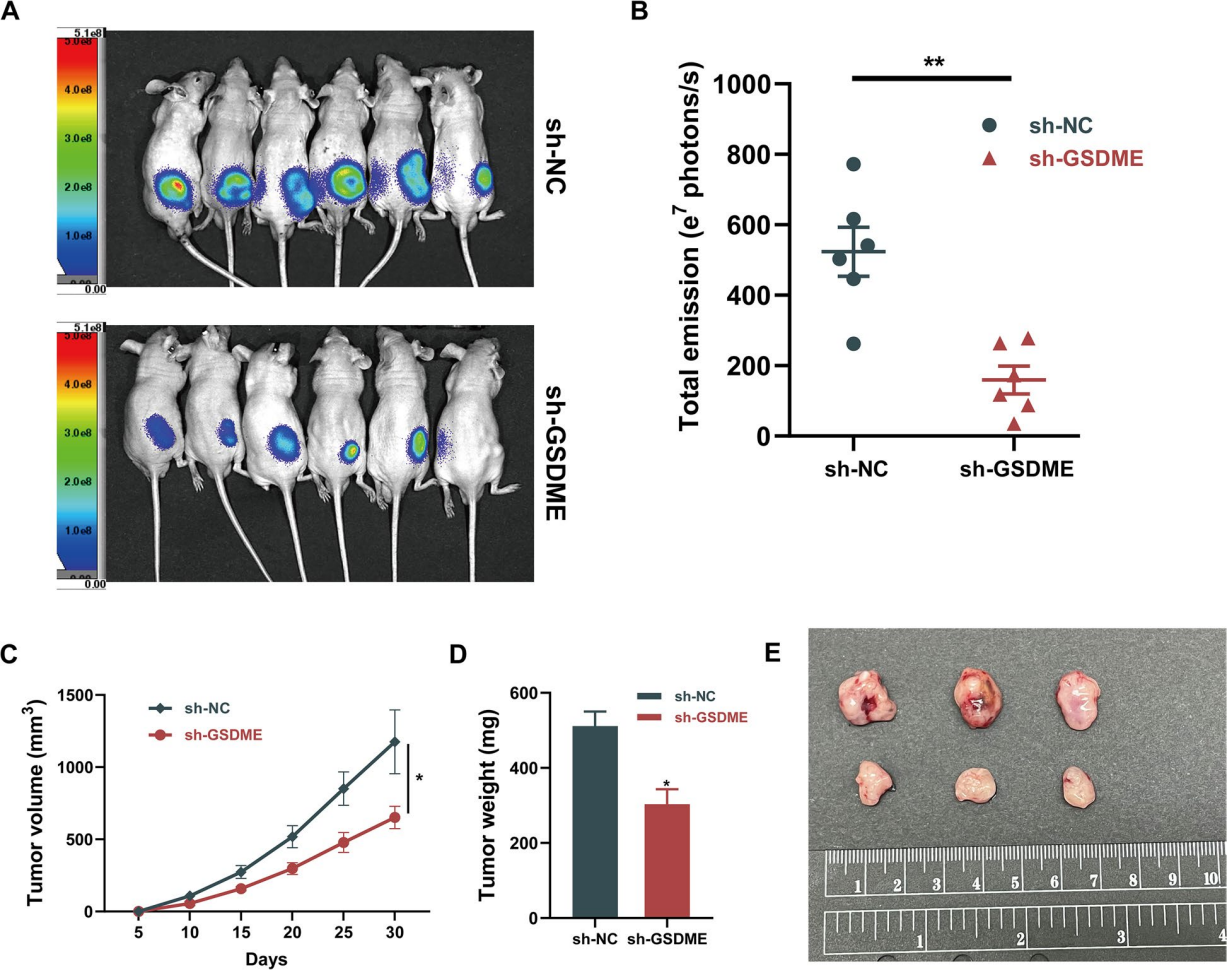
GSDME deficiency reduces the growth of hepatocellular carcinoma in vivo. **A** In vivo fluorescence imaging and **(B)** its quantitative analysis of the tumors that developed in nude mice injected subcutaneously with sh-NC or sh-GSDME SK-Hep1 cells (*n* = 6) on day 30. **C** Tumor volume and **D** tumor xenograft weight was assessed. **E** The xenografts were dissected from the subcutaneous tissues of the mice on day 30 after subcutaneous injection. The data were expressed as means ± SEM. *p < 0.05, **p < 0.01

Additionally, the xenograft models were constructed to assess the role of GSDME on tumor growth in vivo, by injecting SK-Hep1 cells into nude mice following transfection with sh-NC or sh-GSDME. Subcutaneous xenograft tumors implanted into nude mice were monitored with an in vivo imaging system. As shown in Fig. 13A, B, in vivo bioluminescence imaging suggested that the size of the tumor derived from the sh-GSDME group was clearly smaller than those derived from the sh-NC group. Our results also showed that both the size and the weight of tumor xenografts were decreased in the sh-GSDME injected group (Fig. 13C, D). Moreover, tumor xenografts isolated from the nude mice were markedly smaller in the sh-GSDME group (Fig. 13E). Collectively, our data strongly suggest that GSDME knockdown reduces the growth of HCC in vivo. These in vivo results are consistent with those obtained in vitro and confirm that GSDME modulates tumor growth in HCC cells.

### Immune characteristics

**Fig. 14:**
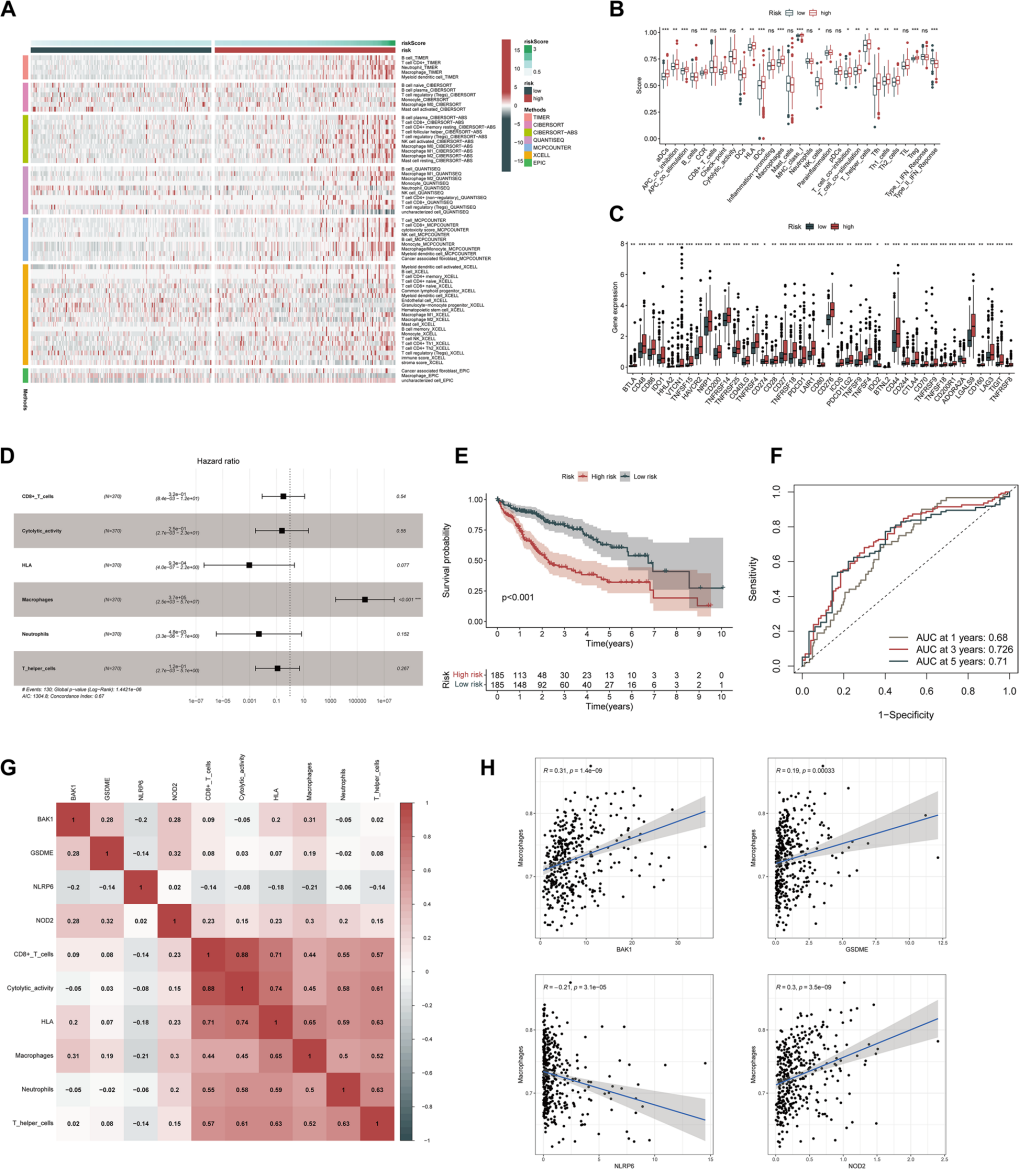
Immune characteristics analysis. **A** Based on TIMER, CIBERSORT, CIBERSORT − ABS, QuanTIseq, MCPcounter, xCell and EpiC algorithms, heatmap of immune infiltration in the high- and low-risk groups. **B** ssGSEA for the association between immune cell subpopulations and related functions. **C** Expression of immune checkpoints among high- and low-risk groups. **D** A multivariate Cox regression analysis of the prognostic value of immune cells and immune-related pathways based on ssGSEA algorithm. **E** Kaplan–Meier survival curve of the OS in the high-/low-risk group. **F** ROC curves for the predicting OS at 1, 3 and 5 years. **G** A heatmap illustrating the co-expressed immune-related signature and PRGs prognostic signature. **H** Associations between each biomarker in the PRGs prognostic signature and Macrophages. Pearson correlation coefficient (R) and its p-value are shown. *p < 0.05, **p < 0.01, ***p < 0.001

The heatmap depicting the immune responses based on EpiC, xCell, MCPcounter, QuanTIseq, CIBERSORT − ABS, CIBERSORT, and TIMER, is presented in Fig. 14A. These findings illustrated that patients having high riskScore exhibited an accumulation of tumor-infiltrating immune cells, including T cell regulatory (Tregs), Macrophage M2, T cell CD4 + , as well as cancer-related fibroblast. The results of each analysis are detailed in Supplementary Table S2. Furthermore, to subsequently investigate the association between immune status and the risk score, we quantitatively analyzed the degree of immunological infiltration about 16 immune cells and the activity of 13 immune-related pathways between the low- and high-risk groups through ssGSEA. Interestingly, the immune cell subsets and related function of Macrophages, Type II IFN Response, Treg, MHC class I, Check-point, APC co-stimulation, aDCs, iDCs, and CCR were markedly different among the two groups (***p < 0.001, Fig. 14B). Given the essentiality that immune checkpoint inhibitors (ICIs) attribute to immunotherapies, differences in the expression of immune checkpoints across the two groups were what we next focused on. We did observe significant differences in the levels of CD274 (PD-L1), PDCD1 (PD-1), LAG3, TIGIT, CTLA4, etc. among others between the two groups (Fig. 14C). Then, immune cells and immune-related pathways based on ssGSEA algorithm were included into multivariate Cox regression analysis to construct an immune-related prognostic model (Fig. 14D). Both the Kaplan–Meier curve and ROC curve indicated excellent prognostic capacity of the immune-related prognostic signature (Fig. 14E, F). Moreover, significant co-expression patterns between immune-related prognostic and PRGs prognostic signature were evaluated by Pearson correlation analysis (Fig. 14G). Of them, there was a highly significant relationship between each biomarker in the PRGs prognostic signature and macrophages (Fig. 14H), and these trends for association were all consistent with our findings mentioned above.

## Discussion

HCC is among the deadliest malignant tumors at a global scale, owing to its complicated and heterogeneous pathogenesis and it is marked by high fatality and recurrence rates, as well as unfavorable prognosis outcomes [1]. The lack of specific biomarkers for diverse tumor types or progression stages addresses a crucial gap in the current understanding and treatment of HCC. In recent years, programmed cell death (PCD) has received increasing attention. Zou et al. investigated the association between twelve PCD patterns (apoptosis, necroptosis, pyroptosis, ferroptosis, cuproptosis, entotic cell death, netotic cell death, parthanatos, lysosome-dependent cell death, autophagy-dependent cell death, alkaliptosis, and oxeiptosis) and tumor prognosis [42]. In the current study, Pyroptosis plays a role in the incidence and progression of various illnesses, playing a dual function in tumor growth by possessing both tumor-promoting and tumor-suppressive characteristics [12]. In detail, we can attenuate tumor load through induction of cancer cell pyroptosis; contrastingly, pyroptosis tends to provoke the release of inflammatory contents, thus resulting in an inflammatory microenvironment of tumor and offering an environmental niche that supports tumorigenesis [43]. Meanwhile, pyroptotic death occurring in tumors could make pyroptosis a suitable target for therapeutic intervention in cancer [44]. However, the carcinogenic mechanism and prognostic value of PRGs in LIHC remain to be elucidated. Thus, in this research, the expression of 52 PRGs in LIHC and their underlying link with OS was systematically investigated. To assess the prognostic significance of these PRGs for LIHC patients, we generated a 4-gene risk model and validated it in the external datasets.

First of all, we identified PRGs that were differently expressed between LIHC and adjoining non-tumor liver tissues in the TCGA by means of examining the gene expression matrix. The differentially expressed PRGs were extracted to conduct GO term and KEGG pathway enrichment analysis, the results of which demonstrated that the majority of enriched genes were in pathways of necroptosis, apoptotic-related processes, and NOD-like receptor signaling pathways. Next, multivariate and univariate Cox regression analyses were performed, eventually identifying four PRGs, namely BAK1, GSDME, NLRP6, and NOD2, that could contribute to assessing the prognosis of LIHC patients. Therefore, based on these four prognosis-related PRGs, we constructed a PRGs-related prognostic risk model for each LIHC patient. Of these four prognostic-associated PRGs, the expression level of NLRP6 was remarkably lowered in high-risk LIHC patients, which was opposite to the changing trend of the remaining three PRGs (BAK1, GSDME, and NOD2). Additionally, the AUC of the ROC curve revealed that the risk signature had satisfactory predictive efficiency in survival prediction. Such a result might be related to the role of these identified PRGs in tumor biology. To illustrate, GSDME, a critical protein in the pyroptosis pathway, is usually expressed at high levels in normal tissues [45], but the abnormally elevated expression could occur in some tumors, including lung cancer [46], gastric carcinoma [47] and melanoma [48]. There are some studies demonstrated that dysregulated GSDME might result in the onset and progression of varieties of human diseases, including malignancies in particular [49]. The study conducted by Zhang et al. indicated that miltirone repressed HCC cells proliferation through BAX-caspase-GSDME-dependent pyroptosis, and the modulation of pyroptosis involved ROS-MEK-ERK1/2 pathway [50]. A similar previous study performed by Yu et al. suggested that GSDME could mediate lobaplatin-mediated pyroptosis implicating the caspase-3/-9 activation and ROS/JNK/Bax-mitochondrial apoptosis pathway [51]. Some classical antitumoral drugs have also been found to exert an effect on tumor immunity by regulating GSDME [52]. Thus, these studies suggested that GSDME might influence some mechanisms in the course of cancer progression and showed the promise of serving as cancer biological markers. Notably, the results from our analyses were in line with a previous research report demonstrating that NLRP6 could suppress inflammation and carcinogenesis in certain tumors such as colorectal carcinogenesis [53]. The role as a tumor suppressor of NLRP6 has also been verified by Wang et al., which indicated that NLRP6 could interact with GRP78 and mediate the breakdown in gastric cancer, thus suppressing the tumorigenesis [54]. Moreover, it is well established that BAK1, a well-known pro-apoptotic regulator, is involved in various biochemical activities [55]. Earlier studies have shown that BAK1 is overexpressed in gastric cancer and related to induction of p53-independent apoptosis [56]. What is more, previous research has shown that BAK1 is upregulated in HCC cells, which might contribute to the tumorigenesis of HCC through ZBP-89 and Sp1 overexpression [57]. A recent study conducted by Zhou et al. revealed that hepatic NOD2 could promote hepatocarcinogenesis through a RIP2-mediated proinflammatory response [58]. NOD2 has also been involved in hepatic inflammation diseases, as attested by its role in promoting hepatitis through inflammatory cytokines production [59], and it is common knowledge that hepatic inflammation is an important contributing factor to the development of HCC. Overall, our findings are in line with several previously published studies.

To further validate the general applicability of our prognostic model, there was a need for stratification analysis utilizing integrating multiple clinical-pathological features. The prognostic model was capable of classifying LIHC patients into high- and low-risk groups accurately, and stratification analysis illustrated that the patients belonging to the high-risk group exhibited remarkably worse prognosis status as opposed to those belonging to the low-risk group in different subgroups stratified by clinical parameters. Meanwhile, GSEA revealed that the oncological characteristics exhibited a significant enrichment in the group with high risks, such as pathways in cancer, cytosolic DNA-sensing pathway, cell cycle, ErbB signaling pathway, NOD-like receptor signaling pathway, and VEGF signaling pathway. There are research reports indicating that cytosolic DNA sensing is involved in the release of immunomodulatory cytokines and contributes, importantly to tumor progression [60, 61]. In addition, the potential targets in our predictive model as GSDME, NPLR6, and NOD2, play a role in the NOD-like receptor signaling pathway, which plays a vital role in pyroptosis [62]. These results may explain, in part, the potential molecular mechanisms of how these potential biomarkers affect prognosis.

Additionally, further verifications by using external cohorts would be required to assess the predictive accuracy of our prognostic signature. We subsequently validated the expression levels of the above-mentioned PRGs in GSE62232, GSE102079, GSE112790, and ICGC cohorts. Moreover, we obtained overall survival analysis data from ICGC and Kaplan–Meier Plotter databases. Considering all these results from external validations, which showed consistent agreement with our results, we further illustrated the independent prognostic value of these above-mentioned PRGs for LIHC. Both in vitro and in vivo experiments also indicated that GSDME knockdown may attenuate proliferation and migration of HCC cells.

Tumor microenvironment (TME) appears to play an essential role in tumorigenesis and progression. Particularly, TME contains several types of tumor‐infiltrating immune cells that have important predictive value for the efficacy of immunotherapy [23]. To further explore potential mechanisms underlying this risk signature in LIHC prognosis, several immune-associated analyses such as EpiC, QuanTIseq, CIBERSORT − ABS, MCPcounter, CIBERSORT, xCell, and TIMER were performed. These findings suggested that patients belonging to the high-risk group accumulated immunosuppressive cells such as Macrophage M2, T cell regulatory (Tregs), T cell CD4 + , and cancer-associated fibroblast. Further research findings indicated that patients belonging to the high-risk group experienced an elevated expression level of immune checkpoint blockade–related genes such as LAG3, PDCD1 (PD-1), CTLA4, CD274 (PD-L1), and TIGIT. Macrophages infiltrated in TME have already been widely considered to induce immunosuppressive microenvironments and ICIs resistance [63], which is generally associated with poor prognosis and adverse outcomes. The prevailing view is that Tregs are viable immunosuppressive cells and perform a fundamental function in tumor progression, immune evasion, and immune tolerance [64]. Intra-tumoral high abundance of Tregs represents a substantial barrier for cancer immunotherapy since it suppresses the anti-tumor response. Lymphocyte-activation gene 3 (LAG3), which is a member of the immunoglobulin superfamily, is reported to be an inhibitory checkpoint receptor that was expressed on activated CD8 + T-cells and impairs its antitumor activity [65], with well-defined an immunosuppressive factor [66]. LAG3 and PD-1 were previously shown to be co-expressed in chronic lymphocytic leukemia antigen-specific CD8 + T cells, contributing to the dysfunctionality of CD8 + T cells [67]. Also, greater expression levels of frequently utilized immune checkpoints associated with immuno-suppressions (including TIGIT, CTLA-4, and PD-L1) could lead to an unfavorable prognosis in patients belonging to the high-risk subgroup. Therefore, the PRGs prognostic signature can be used as a potential immunotherapy target of LIHC.

Although the present research had some meaningful implications, some shortcomings still need to be addressed. First, this study was retrospective, with data extracted from some public databases (TCGA, GEO, and ICGC datasets), thus, validation in a multicenter large sample prospective cohort is warranted. Additionally, although we attempted to determine the prognostic value of GSDME in LIHC through assessment of pathological sections, the results could only reflect the differential expression of GSDME between tumor tissues and adjacent normal tissues. There is, therefore, a need to correlate the protein expression of GSDME with clinical outcomes of LIHC patients. A more significant number of clinical samples with more clinical features-especially long-term clinical outcomes-may be required for the upcoming studies. Second, the existing experimental data was insufficient to explain the mechanism underlying the phenomenon that GSDME likely promotes LIHC tumorigenesis. Hence, future investigation will be required to elucidate the exact molecular mechanism of pyroptosis-associated pathways regulation by GSDME in the progression of LIHC. Thirdly, the expression profile of PRGs, as well as their impact on immunophenotype, were not convincing enough in the absence of in vitro and in vivo tests. In future studies, co-expression network of PRGs and immune-related genes should be given special attention, such as using a double-staining method, and the co-localization between PRGs and immuno-infiltrating cells could be identified using confocal microscopy. Nevertheless, our findings may deliver some evidence and support for future exploration of the possible mechanisms of pyroptosis in patients with LIHC.

## Conclusion

In conclusion, we comprehensively demonstrated the expression, prognostic value, and potential modulation effect on tumor immune infiltration of a four PRGs prognostic signature in LIHC. In addition, we mainly focused on the prognostic value and carcinogenic role of GSDME in LIHC. Our work developed a comprehensive blueprint for the underlying mechanism through which GSDME accelerates the malignant progression of hepatocellular carcinoma and provided novel insights into the development of therapeutic targets as well as potential biomarkers for patients with LIHC.

## Supplementary Information

**Additional file 1: Table S1.** Total 52 pyroptosis-related genes.

**Additional file 2: Supplementary Table S2.** Based on TIMER, CIBERSORT, CIBERSORT−ABS, QuanTIseq, MCPcounter, xCell and EpiC algorithms, heatmap of immune infiltration in the high- and low-risk groups.

**Additional file 3: Figure S1.** Kaplan-Meier survival curves of the 8 significant PRGs screened out by univariate Cox analysis.

**Additional file 4.**

## Data Availability

All data used in the study can be downloaded from multiple data repositories, including the International Cancer Genome Consortium (ICGC, www.icgc.org; ICGC-LIRI-JP), the Cancer Genome Atlas (TCGA, http://cancergenome.nih.gov/; TGCA-LIHC) and NCBI Gene Expression Omnibus (GEO, http://www.ncbi.nlm.nih.gov/geo/; under accession number GSE62232, GSE102079 and GSE112790).
