# Supplementary material for: Identification and in vitro and in vivo validation of the key role of GSDME in pyroptosis-related genes signature in hepatocellular carcinoma
Source: BMC Cancer. 2023 May 6;23:411. doi: 10.1186/s12885-023-10850-1 (PMC10164321; doi:10.1186/s12885-023-10850-1)
Supplement: Supplementary file 1 — Additional file 1: Table S1. Total 52 pyroptosis-related genes. [file 12885_2023_10850_MOESM1_ESM.docx]

**Table S1. Total 52 pyroptosis-related genes.**

| **Genes** | **Full-names** |
| --- | --- |
| BAK1 | BCL2 antagonist/killer 1 |
| BAX | BCL2-associated X |
| CASP1 | cysteine-aspartic acid protease-1 |
| CASP3 | cysteine-aspartic acid protease-3 |
| CASP4 | cysteine-aspartic acid protease-4 |
| CASP5 | cysteine-aspartic acid protease-5 |
| CHMP2A | charged multivesicular body protein 2A |
| CHMP2B | charged multivesicular body protein 2B |
| CHMP3 | charged multivesicular body protein 3 |
| CHMP4A | charged multivesicular body protein 4A |
| CHMP4B | charged multivesicular body protein 4B |
| CHMP4C | charged multivesicular body protein 4C |
| CHMP6 | charged multivesicular body protein 6 |
| CHMP7 | charged multivesicular body protein 7 |
| CYCS | cytochrome C |
| ELANE | elastase, neutrophil expressed |
| GSDMD | gasdermin D |
| GSDME | gasdermin E |
| GZMB | granzyme B |
| HMGB1 | high mobility group box 1 |
| IL18 | interleukin 18 |
| IL1A | interleukin 1 alpha |
| IL1B | interleukin 1 beta |
| IRF1 | interferon regulatory factor 1 |
| IRF2 | interferon regulatory factor 2 |
| TP53 | tumor protein p53 |
| TP63 | tumor protein p63 |
| AIM2 | absent in melanoma 2 |
| CASP6 | cysteine-aspartic acid protease-6 |
| CASP8 | cysteine-aspartic acid protease-8 |
| CASP9 | cysteine-aspartic acid protease-9 |
| GPX4 | glutathione peroxidase 4 |
| GSDMA | gasdermin A |
| GSDMB | gasdermin B |
| GSDMC | gasdermin C |
| IL6 | interleukin 6 |
| NLRC4 | NLR family CARD domain containing 4 |
| NLRP1 | NLR family pyrin domain containing 1 |
| NLRP2 | NLR family pyrin domain containing 2 |
| NLRP3 | NLR family pyrin domain containing 3 |
| NLRP6 | NLR family pyrin domain containing 6 |
| NLRP7 | NLR family pyrin domain containing 7 |
| NOD1 | nucleotide binding oligomerization domain containing 1 |
| NOD2 | nucleotide binding oligomerization domain containing 2 |
| PJVK | pejvakin |
| PLCG1 | phospholipase C gamma 1 |
| PRKACA | protein kinase cAMP-activated catalytic subunit alpha |
| PYCARD | PYD and CARD domain containing |
| SCAF11 | SR-related CTD associated factor 11 |
| TIRAP | TIR domain containing adaptor protein |
| TNF | tumor necrosis factor |
| GZMA | granzyme A |
