# Supplementary material for: Identification and in vitro and in vivo validation of the key role of GSDME in pyroptosis-related genes signature in hepatocellular carcinoma
Source: BMC Cancer. 2023 May 6;23:411. doi: 10.1186/s12885-023-10850-1 (PMC10164321; doi:10.1186/s12885-023-10850-1)
Supplement: Supplementary file 2 — Additional file 2: Supplementary Table S2. Based on TIMER, CIBERSORT, CIBERSORT−ABS, QuanTIseq, MCPcounter, xCell and EpiC algorithms, heatmap of immune infiltration in the high- and low-risk groups. [file 12885_2023_10850_MOESM2_ESM.docx]

| **Supplementary Table S2. Based on TIMER, CIBERSORT, CIBERSORT−ABS, QuanTIseq, MCPcounter, xCell and EpiC algorithms, heatmap of immune infiltration in the high- and low-risk groups.** | |
| --- | --- |
| **Immune** | **P-value** |
| Macrophage_EPIC | 9.01E-16 |
| uncharacterized cell_EPIC | 1.81E-14 |
| Macrophage M0_CIBERSORT-ABS | 2.42E-14 |
| Myeloid dendritic cell_TIMER | 8.73E-13 |
| Macrophage_TIMER | 1.36E-12 |
| Monocyte_MCPCOUNTER | 6.24E-12 |
| Macrophage/Monocyte_MCPCOUNTER | 6.24E-12 |
| T cell CD4+ Th2_XCELL | 6.93E-12 |
| T cell regulatory (Tregs)_QUANTISEQ | 1.85E-11 |
| Common lymphoid progenitor_XCELL | 2.66E-11 |
| Endothelial cell_XCELL | 3.85E-11 |
| Macrophage M0_CIBERSORT | 7.43E-10 |
| stroma score_XCELL | 1.68E-09 |
| B cell_TIMER | 2.75E-09 |
| Neutrophil_TIMER | 3.43E-09 |
| B cell_MCPCOUNTER | 5.20E-09 |
| T cell_MCPCOUNTER | 5.94E-09 |
| T cell CD4+_TIMER | 6.26E-09 |
| B cell_QUANTISEQ | 1.58E-08 |
| Macrophage M2_CIBERSORT-ABS | 5.10E-08 |
| T cell CD8+_QUANTISEQ | 5.90E-08 |
| T cell regulatory (Tregs)_CIBERSORT-ABS | 8.89E-08 |
| Hematopoietic stem cell_XCELL | 9.10E-08 |
| NK cell activated_CIBERSORT-ABS | 1.05E-06 |
| Myeloid dendritic cell_MCPCOUNTER | 1.61E-06 |
| T cell follicular helper_CIBERSORT-ABS | 4.11E-06 |
| Cancer associated fibroblast_EPIC | 1.33E-05 |
| Monocyte_QUANTISEQ | 2.16E-05 |
| B cell_XCELL | 4.35E-05 |
| T cell CD8+ naive_XCELL | 0.000132874 |
| Granulocyte-monocyte progenitor_XCELL | 0.000194133 |
| B cell plasma_CIBERSORT-ABS | 0.000273993 |
| T cell NK_XCELL | 0.000298402 |
| Monocyte_CIBERSORT | 0.000499617 |
| NK cell_QUANTISEQ | 0.000521248 |
| T cell regulatory (Tregs)_XCELL | 0.00054719 |
| Macrophage M1_QUANTISEQ | 0.000819477 |
| T cell CD8+_MCPCOUNTER | 0.000950395 |
| Macrophage M2_XCELL | 0.00104154 |
| T cell CD4+ memory_XCELL | 0.001242972 |
| Macrophage M1_CIBERSORT-ABS | 0.001804869 |
| T cell CD4+ (non-regulatory)_QUANTISEQ | 0.002082809 |
| Mast cell activated_CIBERSORT | 0.002592201 |
| Cancer associated fibroblast_MCPCOUNTER | 0.002786815 |
| uncharacterized cell_QUANTISEQ | 0.003826307 |
| cytotoxicity score_MCPCOUNTER | 0.005006917 |
| Myeloid dendritic cell_XCELL | 0.005425508 |
| Macrophage M1_XCELL | 0.005940991 |
| T cell CD4+ naive_XCELL | 0.006304372 |
| T cell CD4+ memory resting_CIBERSORT-ABS | 0.006559806 |
| Macrophage M2_QUANTISEQ | 0.006701629 |
| Myeloid dendritic cell activated_XCELL | 0.007346188 |
| T cell regulatory (Tregs)_CIBERSORT | 0.007430339 |
| T cell CD8+_CIBERSORT-ABS | 0.008657104 |
| immune score_XCELL | 0.009905639 |
| T cell CD4+ Th1_XCELL | 0.012387271 |
| Neutrophil_QUANTISEQ | 0.013211993 |
| Mast cell_XCELL | 0.015966209 |
| B cell plasma_CIBERSORT | 0.017188467 |
| B cell memory_XCELL | 0.022943182 |
| NK cell_MCPCOUNTER | 0.027329874 |
| Monocyte_XCELL | 0.042102512 |
| B cell naive_CIBERSORT | 0.042563196 |
| Mast cell resting_CIBERSORT-ABS | 0.049190738 |
