# Supplementary figures and images for: Identification and in vitro and in vivo validation of the key role of GSDME in pyroptosis-related genes signature in hepatocellular carcinoma
Source: BMC Cancer. 2023 May 6;23:411. doi: 10.1186/s12885-023-10850-1 (PMC10164321; doi:10.1186/s12885-023-10850-1)

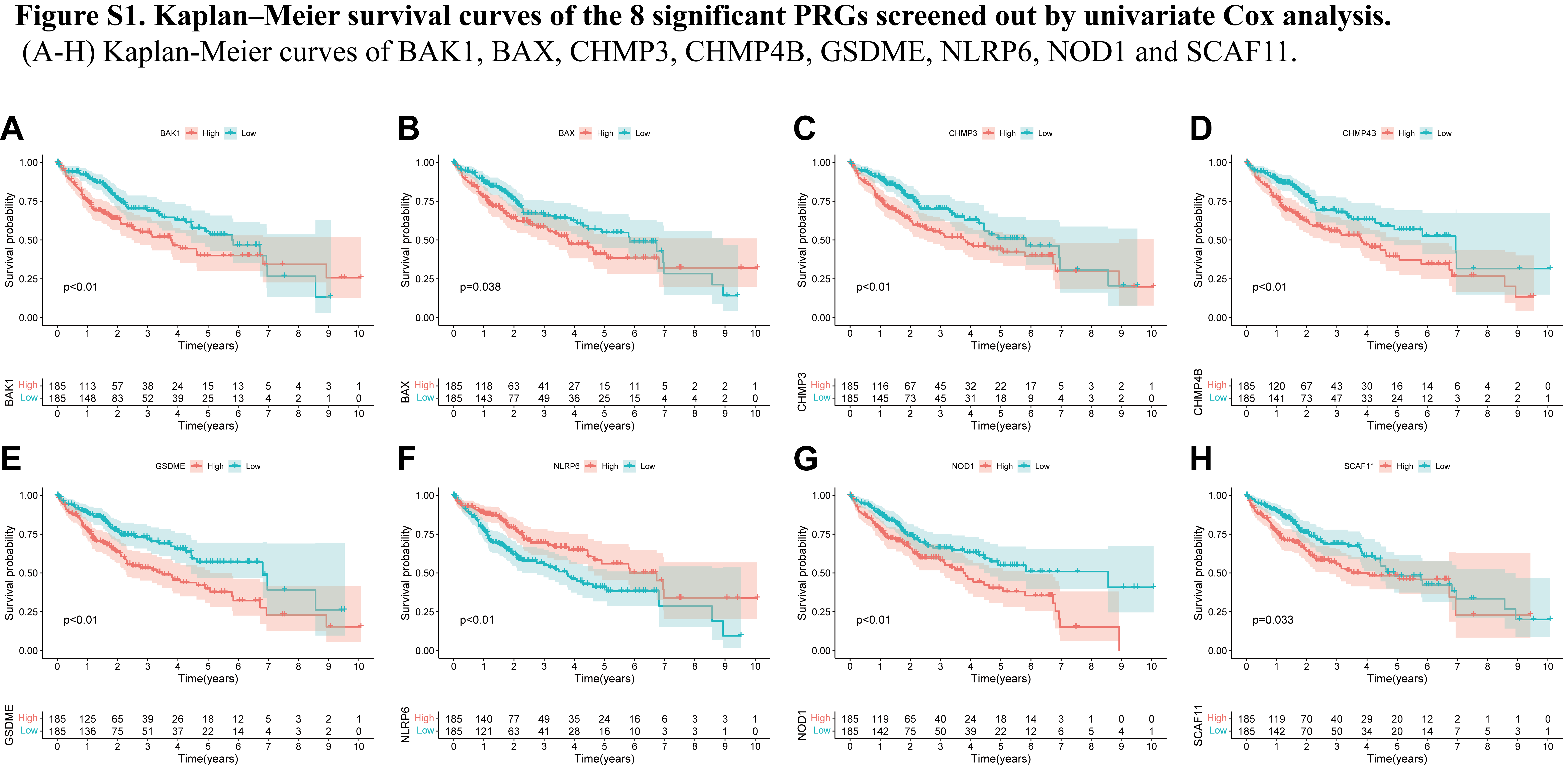

Supplement: Supplementary file 3 — Additional file 3: Figure S1. Kaplan-Meier survival curves of the 8 significant PRGs screened out by univariate Cox analysis. [file 12885_2023_10850_MOESM3_ESM.tif]

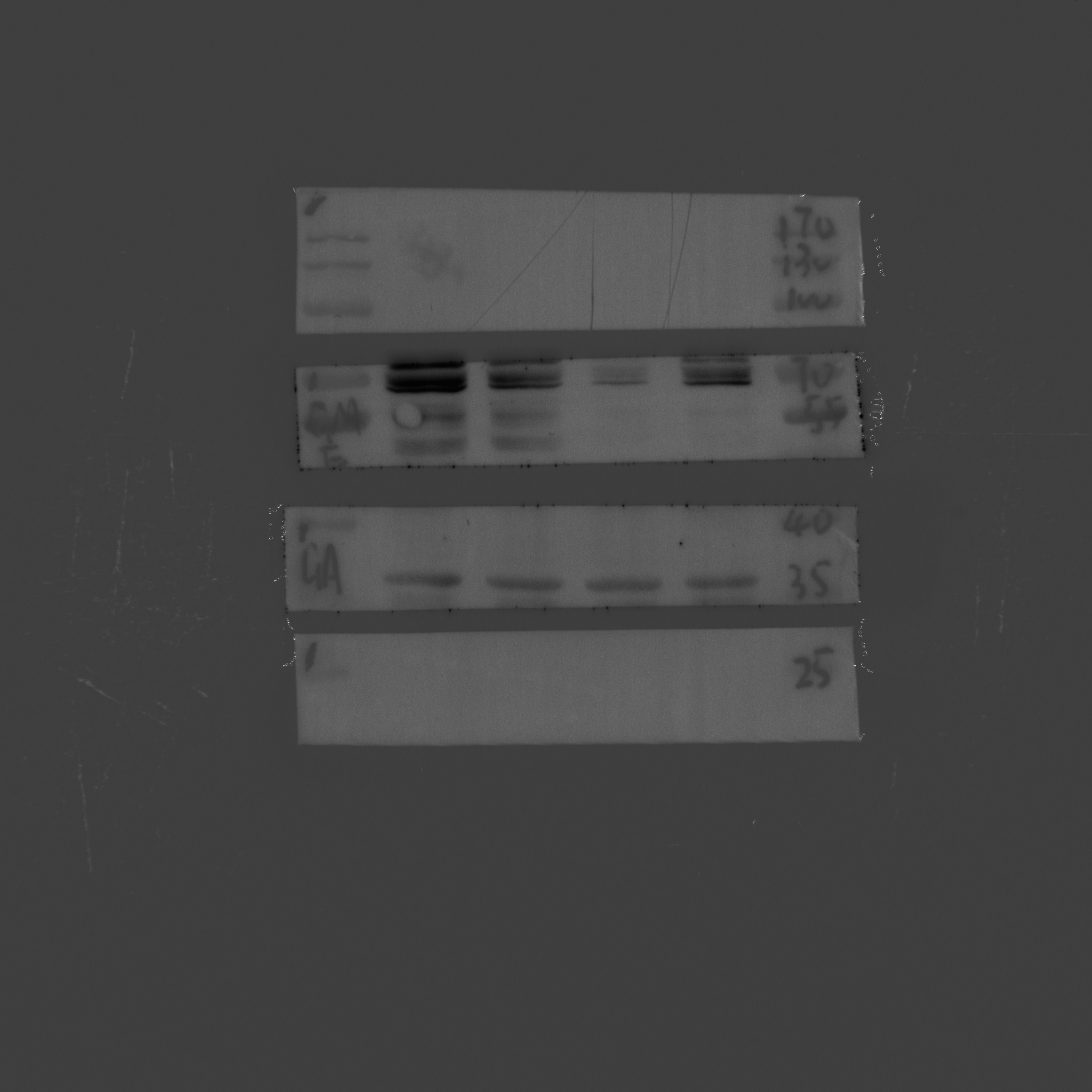

Supplement: Supplementary file 4 — Additional file 4. [file 12885_2023_10850_MOESM4_ESM.zip › Figure 11D.tif]

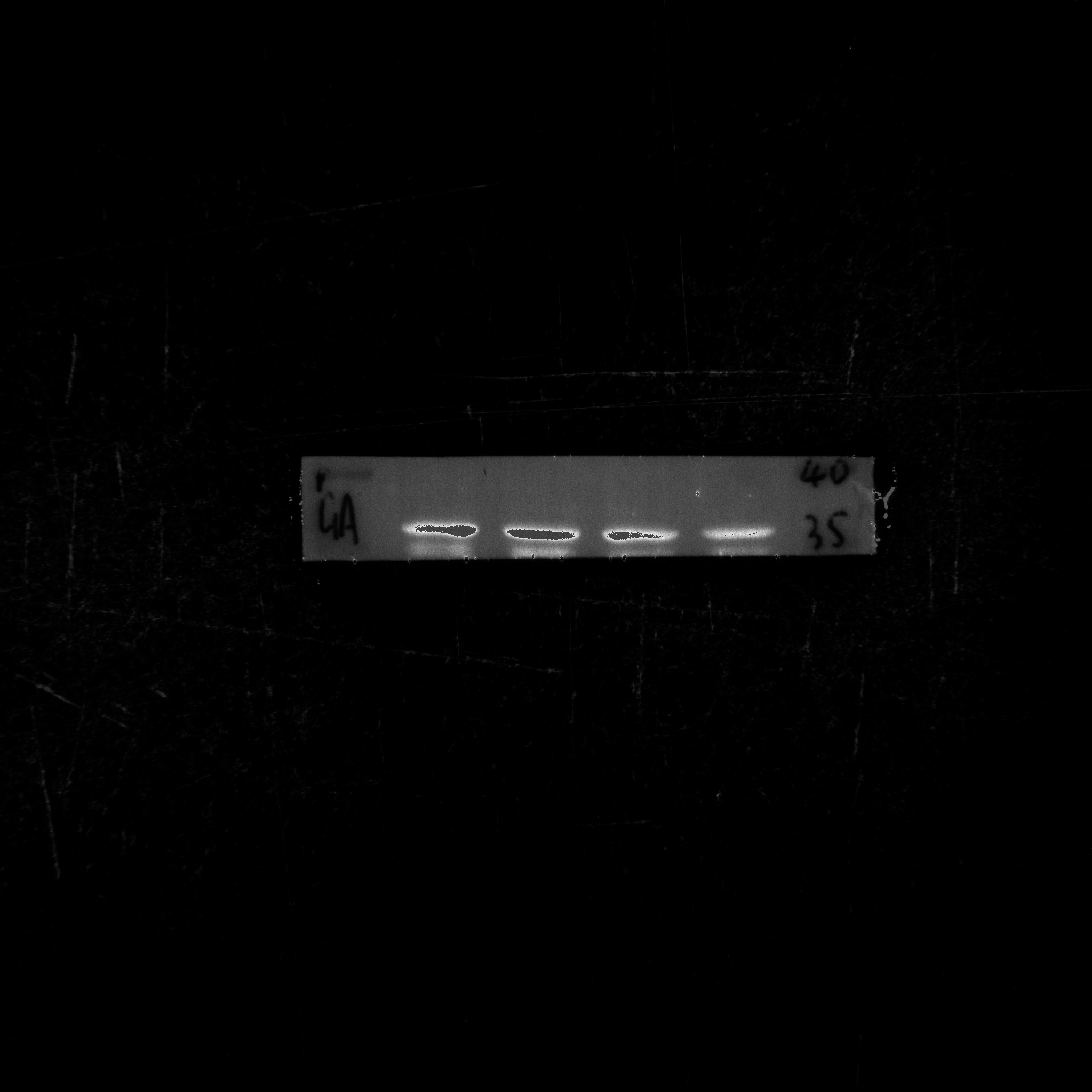

Supplement: Supplementary file 4 — Additional file 4. [file 12885_2023_10850_MOESM4_ESM.zip › Figure 11D-GAPDH.tif]

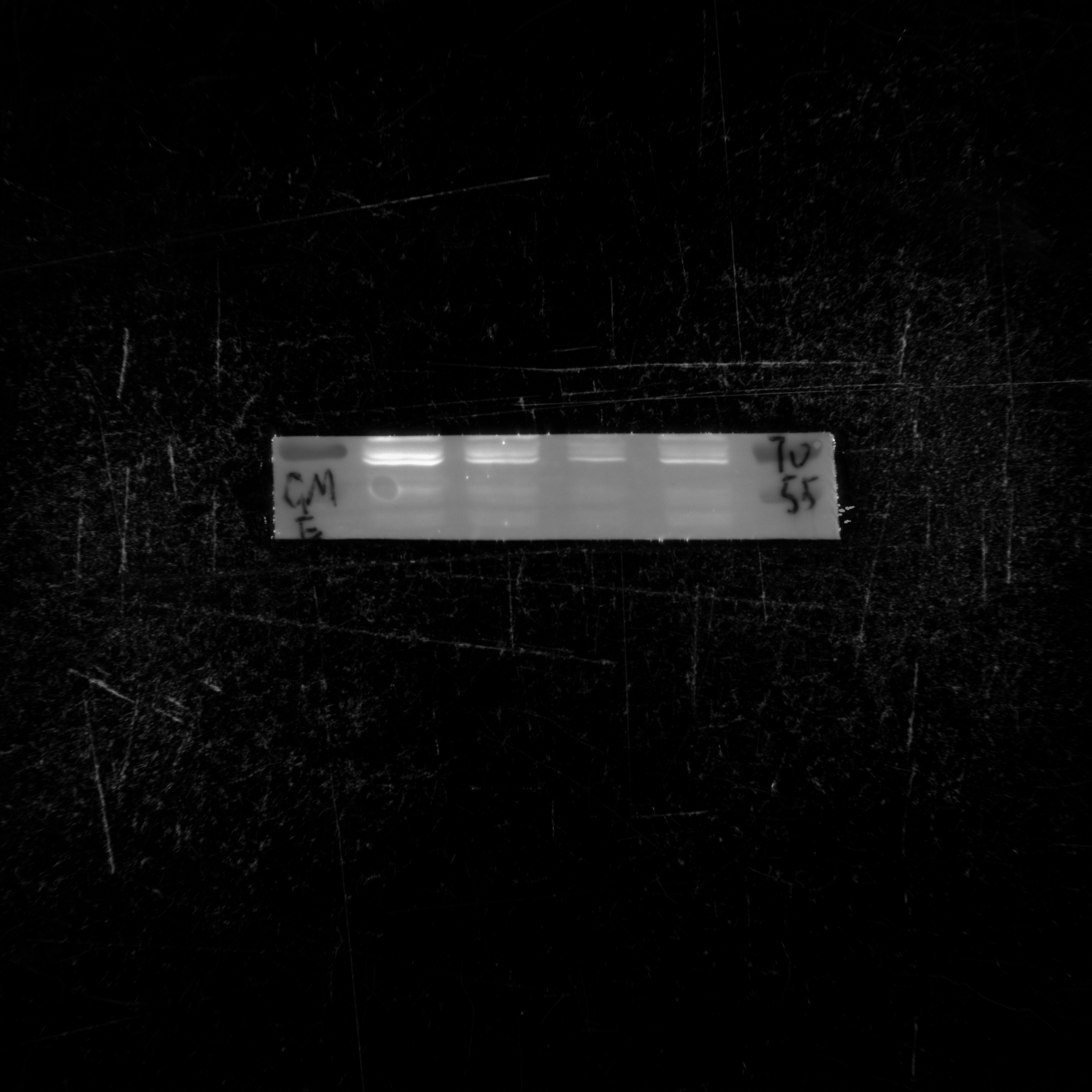

Supplement: Supplementary file 4 — Additional file 4. [file 12885_2023_10850_MOESM4_ESM.zip › Figure 11D-GSDME.tif]

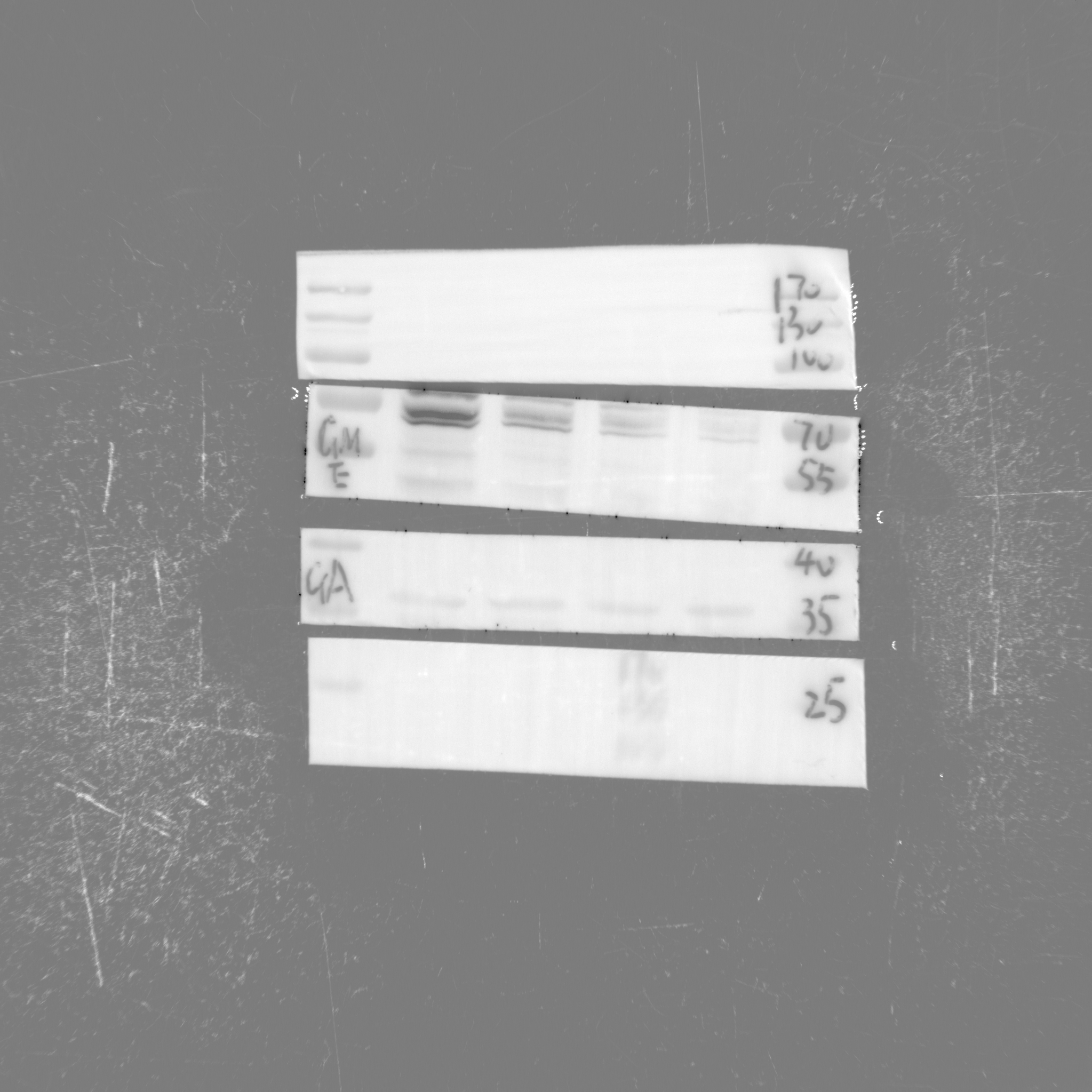

Supplement: Supplementary file 4 — Additional file 4. [file 12885_2023_10850_MOESM4_ESM.zip › Figure 11E(1).tif]

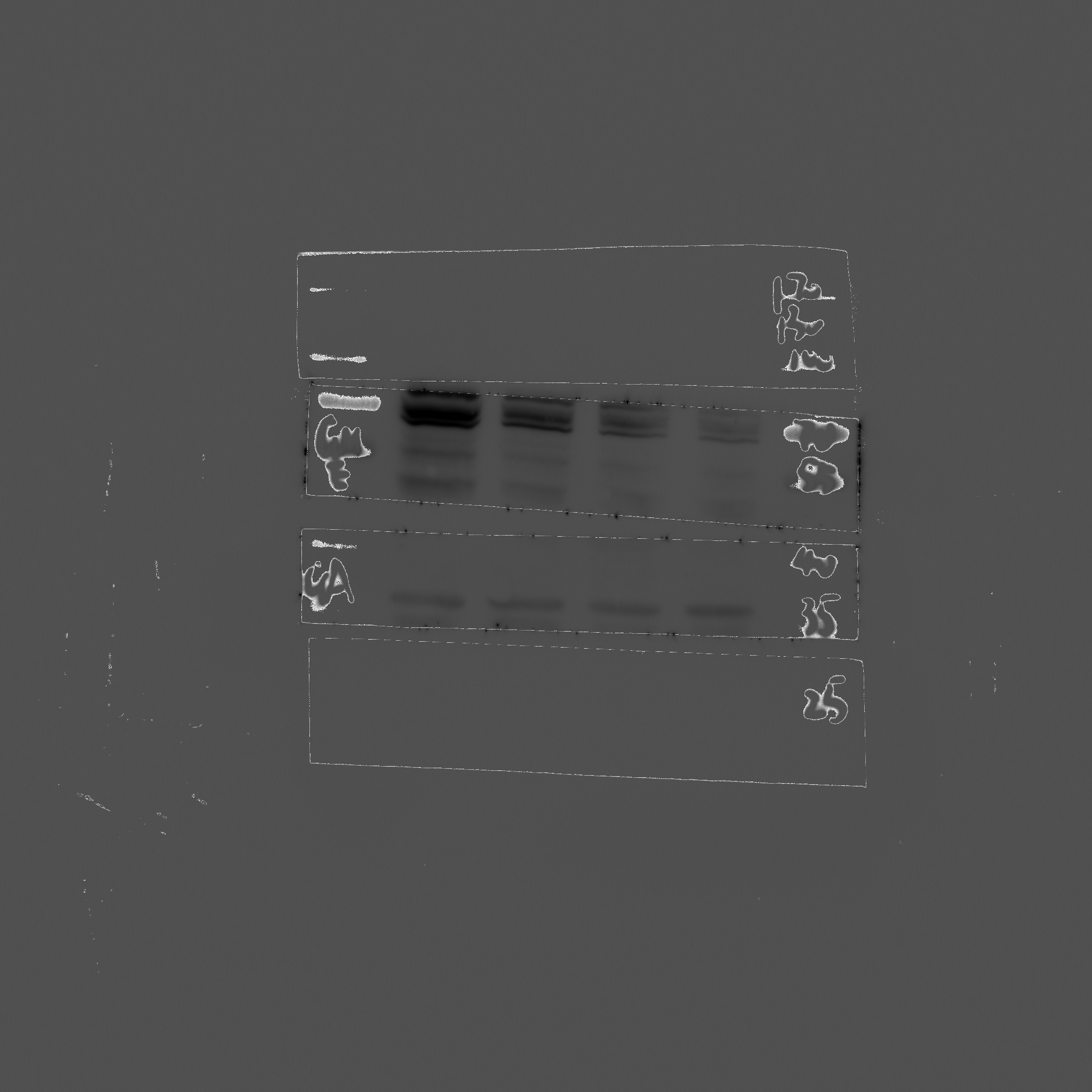

Supplement: Supplementary file 4 — Additional file 4. [file 12885_2023_10850_MOESM4_ESM.zip › Figure 11E.tif]

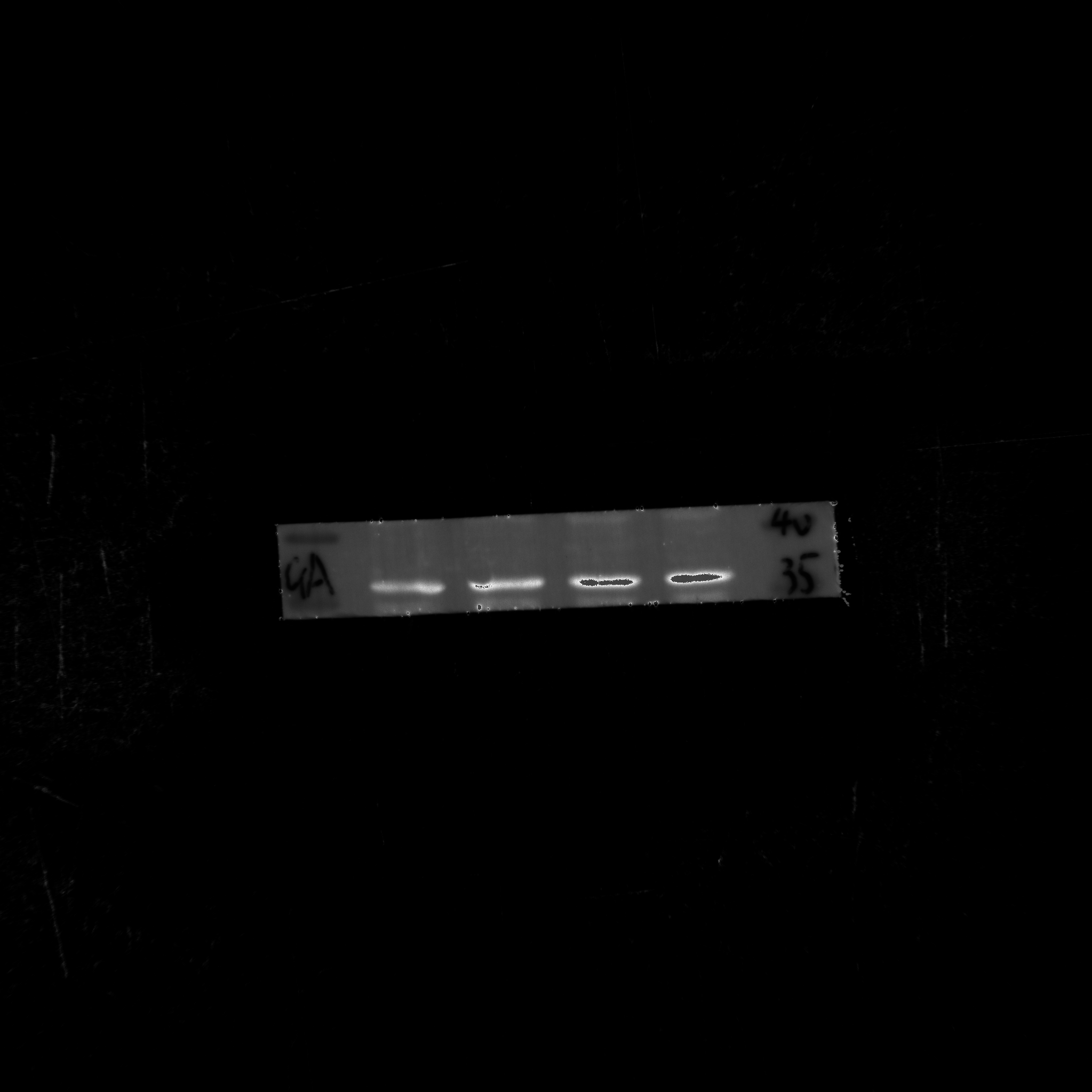

Supplement: Supplementary file 4 — Additional file 4. [file 12885_2023_10850_MOESM4_ESM.zip › Figure 11E-GAPDH.tif]

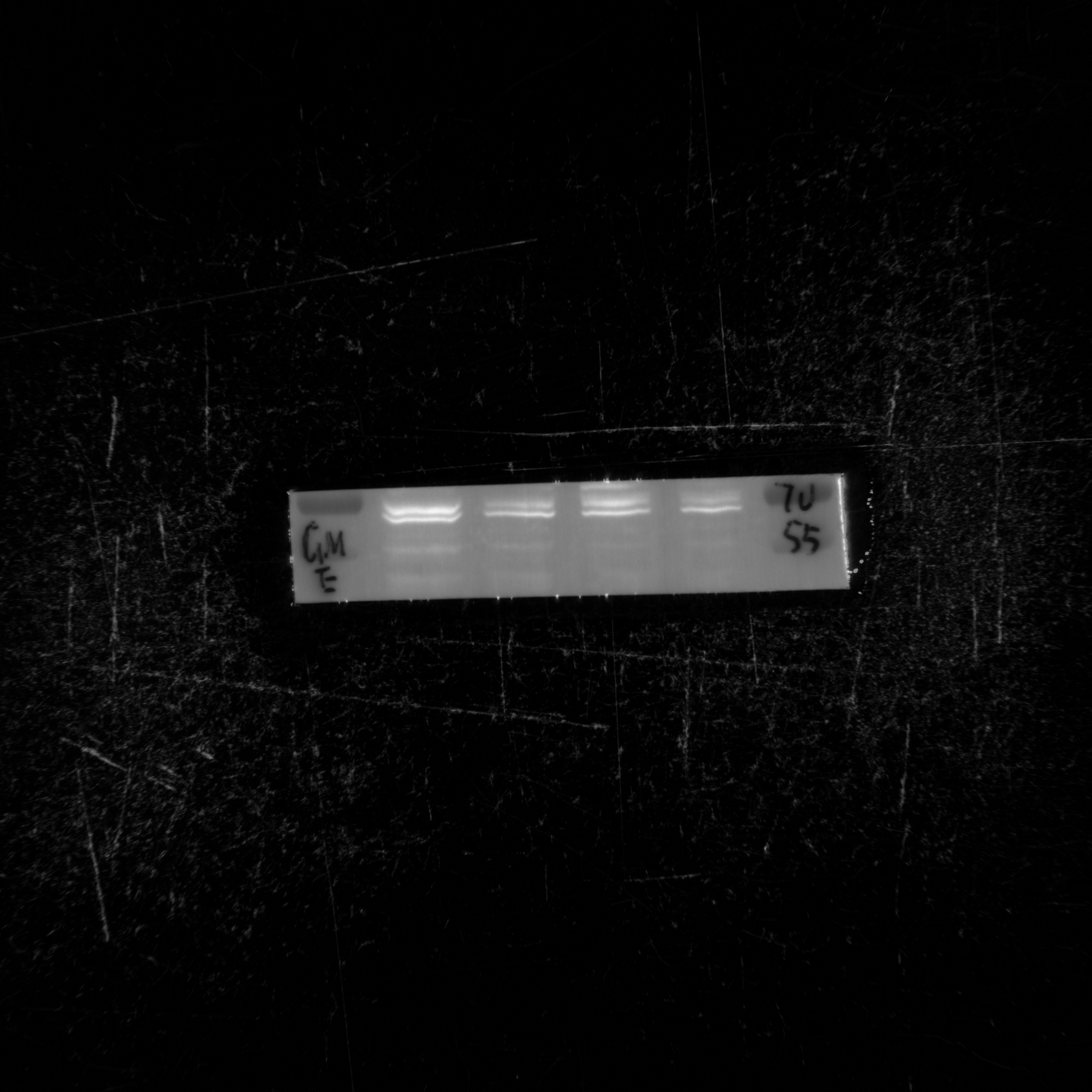

Supplement: Supplementary file 4 — Additional file 4. [file 12885_2023_10850_MOESM4_ESM.zip › Figure 11E-GSDME.tif]
